# Identification of a Resistance Exercise-Specific Signaling Pathway that Drives Skeletal Muscle Growth

**DOI:** 10.21203/rs.3.rs-4997138/v1

**Published:** 2024-11-12

**Authors:** Wenyuan G. Zhu, Aaron CQ Thomas, Gary M Wilson, Jamie E Hibbert, Corey GK Flynn, Chris McGlory, Kent W Jorgenson, Nathaniel D. Steinert, Kuan-Hung Lin, Martin J MacInnis, Joshua J. Coon, Stuart M. Phillips, Troy A. Hornberger

**Affiliations:** 1 Department of Comparative Biosciences, University of Wisconsin-Madison; 2 School of Veterinary Medicine, University of Wisconsin-Madison; 3 Department of Kinesiology, McMaster University. School of Kinesiology and Health Studies; 4 Department of Chemistry, University of Wisconsin-Madison; 5 Department of Biomolecular Chemistry, University of Wisconsin-Madison; 6 National Center for Quantitative Biology of Complex Systems, University of Wisconsin-Madison; 7 Morgridge Institute for Research, University of Wisconsin-Madison; 8 Department of Medicine, Queen’s University.

## Abstract

A human model of unilateral endurance versus resistance exercise, in conjunction with deep phosphoproteomic analyses, was used to identify exercise mode-specific phosphorylation events. Among the outcomes, a resistance exercise-specific cluster of events was identified, and a multitude of bioinformatic- and literature-based predictions suggested that this was mediated by prolonged activation of a pathway involving MKK3b/6, p38, MK2, and mTORC1. Follow-up studies in humans and mice provide consistent support for the predictions and also revealed that resistance exercise-induced signaling through MKK3b and the induction of protein synthesis are highly correlated events (R = 0.87). Moreover, genetic activation of MKK3b/6 in skeletal muscles was sufficient to induce signaling through the members of the resistance exercise-specific pathway, as well as an increase in protein synthesis and fiber size. Thus, we propose that we have identified some of the core components of a signaling pathway that drives the growth-promoting effects of resistance exercise.

## Introduction

Skeletal muscle is a remarkably malleable tissue that can rapidly adapt to changes in functional demands. For instance, endurance exercise can lead to a robust increase in capillary density, mitochondrial content, and a concomitant increase in aerobic capacity but only exerts a minimal effect on the synthesis of myofibrillar proteins, muscle mass, and strength. Conversely, resistance exercise can promote a robust increase in the synthesis of myofibrillar proteins, muscle mass, and strength but exerts little, if any, effect on aerobic capacity ^1,2^. Although the distinct physiological and biochemical adaptations that occur in response to these different modes of exercise are well known, the mechanisms that drive them remain far from defined ^2,3^.

For skeletal muscle to respond with such distinct adaptations, it must be able to sense and transmit the information that is specific to each mode of exercise. Typically, the transmission of such information is mediated by signaling pathways that rely on the use of posttranslational modifications, and one of the most well-recognized posttranslational modifications is phosphorylation ^4^. Indeed, current estimates indicate that more than 75% of all proteins are phosphorylated at some point during their life cycle, and phosphorylation-dependent signaling pathways have been implicated in the regulation of nearly all cellular processes ^5,6^. As such, we reasoned that the identification of phosphorylation events that are differentially regulated by endurance and resistance exercise would enable us to gain insight into the mechanisms that drive the exercise mode-specific adaptations.

## Results

### Identification of the phosphoproteomic alterations that occur after a bout of endurance versus resistance exercise.

**Figure 1.**
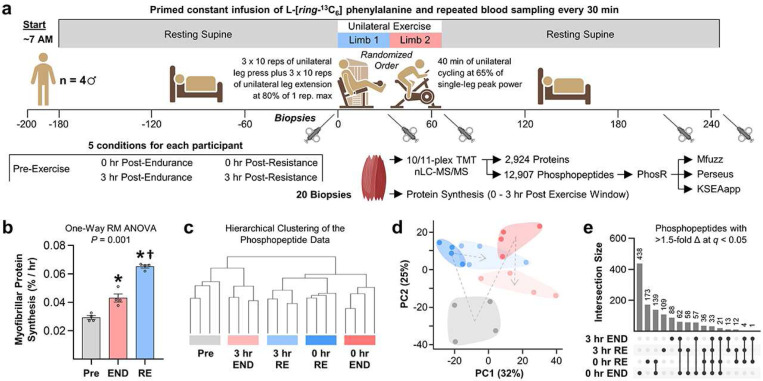
Overview of the phosphoproteomic alterations that occur after a bout of endurance versus resistance exercise in humans **a**, Timeline of the experimental interventions and a general description of the analytical procedures that were employed. **b**, Measurement of the mean rate of myofibrillar protein synthesis during three different 3 hr windows of time including: i) 3 hr pre-exercise (Pre), ii) immediately post (0 hr) to 3 hr post endurance exercise (END), and iii) 0 hr to 3 hr post resistance exercise (RE). Values are group means ± SEM, n = 4 per group. Data were analyzed with one-way repeated measures (RM) ANOVA. * Significantly different from Pre, † significantly different from END, *P* < 0.05. **c**, Unsupervised hierarchical clustering of the phosphopeptide data from each of the participants. **d**, Principal component analysis of the phosphopeptide data from each of the participants. Dots represent the values for each participant and the color of the dot indicates the experimental condition (as described in c). **e**, UpSet plot illustrating the number of phosphopeptides for each condition that experienced a > 1.5-fold exercise-induced change (Δ) in abundance at an FDR-corrected *P*-value (*q*) of < 0.05 when compared with Pre.

To identify phosphorylation events that are differentially regulated by endurance and resistance exercise we employed a human model of unilateral exercise that allowed for a within-subject repeated measure design. Specifically, as illustrated in Figure 1a, four men (age, 21.3 ± 0.3 years; BMI, 26.1 ± 1.3 kg/m^2^; VO_2_ peak, 47.3 ± 1.5 mL/kg/min, Supplementary Table 1) fasted overnight and then one leg was subjected intense unilateral resistance exercise whereas the other was subjected to intense unilateral endurance exercise. The order for performing the different modes of exercise was randomized, and biopsies from the vastus lateralis muscles were collected immediately before the onset of the first exercise bout (Pre-exercise), as well as immediately (0 hr) and 3 hr after the completion of each bout. A portion of the pre-exercise and 3 hr post-exercise biopsies were then analyzed for the rate of protein synthesis and the results indicated that both modes of exercise led to an increase in the synthesis of myofibrillar proteins but, as expected, the effect of resistance exercise was much more robust (Fig. 1b).

**Figure 2.**
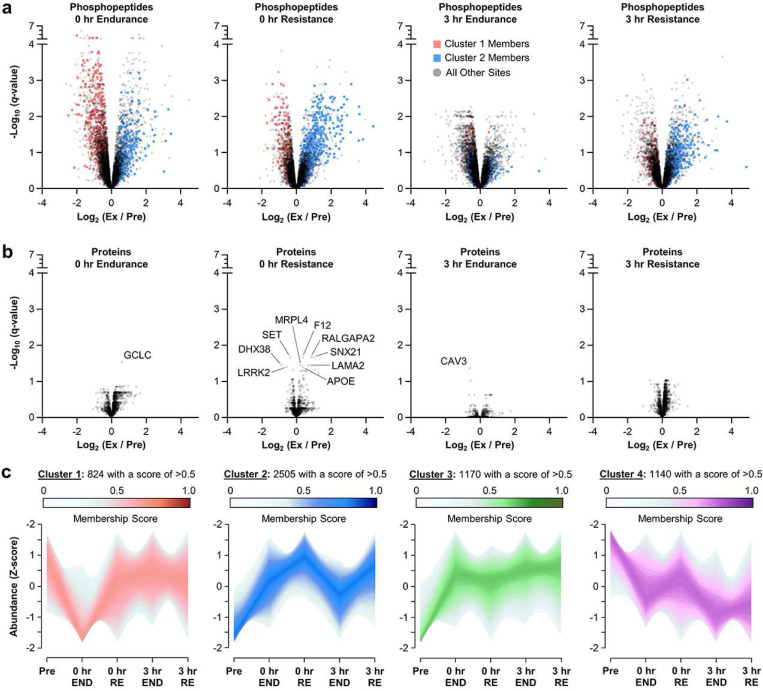
Cluster-based identification of phosphorylation events that are specific to endurance and resistance exercise in humans **a**, Volcano plots from the phosphoproteomic analyses at 0 hr or 3 hr post endurance (END) or resistance (RE) exercise. **b**, Volcano plots from the proteomic analyses for the same conditions listed in **a.** The highlighted proteins experienced a significant alteration in abundance, *q* ≤ 0.05. **c**, Soft clustering of the phosphoproteomic data revealed four major clusters of phosphopeptides that were affected by exercise. In **a**, the phosphopeptides that had a membership score of ≥ 0.5 for cluster 1 are highlighted in pink, while those with a membership score of ≥ 0.5 for cluster 2 are highlighted in blue.

Having observed the expected response at the level of myofibrillar protein synthesis, we next subjected the biopsies to a deep multiplexed proteomic and phosphoproteomic workflow ^7^. In total, the analyses provided quantitative information on 2,924 different proteins and 12,907 unique phosphopeptides (Supplementary Tables 2 and 3, respectively). Further processing of the phosphopeptide data with PhosR revealed that the individual samples clustered according to the mode of exercise and the time-point post-exercise (Fig. 1c) ^8^. Moreover, PCA analysis and UpSet plots revealed large perturbations of the phosphoproteome at 0 hr post endurance exercise and 0 hr post resistance exercise (Fig. 1d,e). However, the direction of the perturbations between the two modes of exercise was different, and the large perturbation of the phosphoproteome was still evident at 3 hr post resistance exercise, whereas a partial return towards the pre-exercise state was observed at 3 hr post endurance exercise (Fig. 1d and 2a). Importantly, the proteome remained largely unperturbed which suggested that the changes at the level of the phosphopeptides were mediated by alterations in the activity of kinases and/or phosphatases (Fig. 2b).

### Detection of phosphorylation events that are specific to endurance and resistance exercise.

To gain further insight into the phosphorylation events that were differentially regulated by endurance and resistance exercise we performed unsupervised soft clustering with mFuzz ^9^. As shown in Fig. 2c, the analysis identified four clusters of phosphopeptides that were affected by exercise, with cluster 1 and cluster 2 showing signs of particular interest. For instance, cluster 1 was marked by the prevalence of phosphopeptides that underwent a decrease in phosphorylation specifically after endurance exercise, whereas cluster 2 was dominated by phosphopeptides that experienced a prolonged elevation in phosphorylation specifically after resistance exercise (Fig. 2a,c).

The exercise mode-specific nature of clusters 1 and 2 was intriguing, and therefore we wanted to develop a better understanding of the cellular effects that might be conferred by the phosphorylation events within each cluster. To accomplish this, the phosphopeptide data was subjected to 1D enrichment analysis ^10^ and, when the cluster 1 membership score was used as the dimensional input, a variety of annotation terms were found to be significantly over- or under-represented. However, annotation terms that could be linked to endurance exercise-specific adaptations were not readily identified (Extended Data Fig. 1a and Supplementary Table 4). On the other hand, when the cluster 2 membership score was used as the dimensional input, a very pronounced over-representation of phosphorylation events on proteins that were annotated with gene ontology terms such as “translation”, and the “positive regulation of skeletal muscle growth” was observed (Extended Data Fig. 1b and Supplementary Table 4).

### Inference of kinases that are differentially regulated by endurance and resistance exercise.

**Figure 3.**
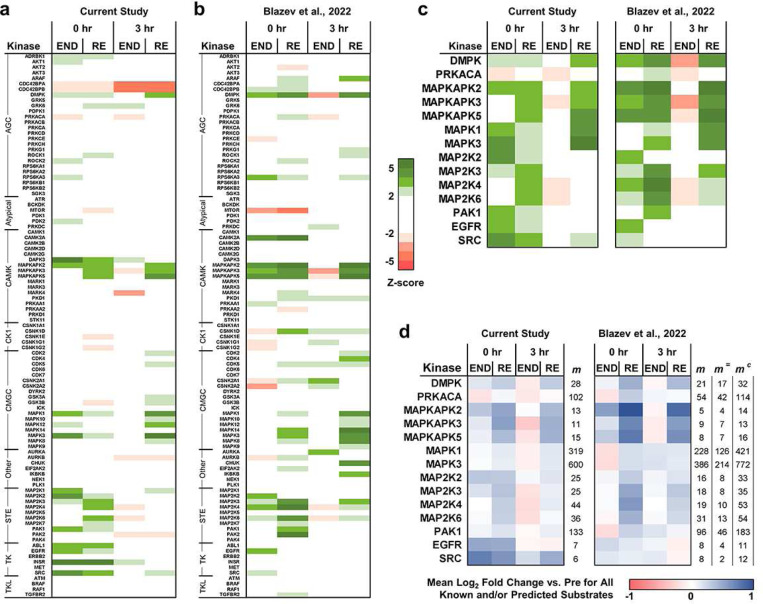
Identification of kinases that are reproducibly inferred as being regulated by endurance and/or resistance exercise in humans **a**, Heatmap of Z-scores for the KSEAapp inferred alterations in kinase activity at 0 hr or 3 hr post endurance (END) or resistance (RE) exercise when compared to the pre-exercise state. Kinases are listed by their gene names and only kinases expressed in skeletal muscle (see methods) that possessed at least 5 known and/or predicted substrates in the phosphopeptide dataset are listed. The list of kinases was clustered according to their group (e.g., AGC) ^68^ and then listed in alphabetical order. **b**, The results from the current study are compared with the results that were derived from identical processing of the phosphopeptide dataset published by Blazev et al., 2022 ^14^. **c**, Heatmaps of the Z-scores for the kinases whose activity was inferred to be significantly altered (*q* ≤ 0.05) in at least one condition in both the Blazev et al., 2022 dataset and the current dataset. **d**, Heatmaps of the mean exercise-induced change in the phosphorylation of the known and/or predicted substrates of each kinase listed in (**c**). Also shown is the number of known and/or predicted substrates for each kinase (m), the number of m that were common to both datasets $\left(m^{=}\right)$, and the combined number of distinct m from the two datasets $\left(m^{c}\right)$.

The results from the 1D enrichment analysis suggested that the phosphorylation events in cluster 2 (i.e., the resistance exercise-specific cluster) might be linked to the signaling pathway that drives the resistance exercise-induced increase in protein synthesis and growth. Thus, to further explore this possibility, the phosphopeptide data in conjunction with KSEAapp ^11^ were used to infer how the different modes of exercise affected the activity of the kinases that are expressed in skeletal muscle. More precisely, KSEAapp uses the phosphorylation state of the substrates for each kinase to generate a Z-score, with a positive Z-score inferring activation of the kinase and a negative Z-score inferring inhibition of the kinase. Importantly, the inferences can be derived exclusively from known kinase-substrate pairs that are listed in PhosphoSitePlus ^12^, or with the addition of predicted kinase-substrate pairs derived from NetworKIN ^13^. In our dataset, there were only 820 known kinase-substate pairs and this limited the statistical power of the inferences that could be made. Therefore, an additional 5,523 predicted kinase-substrate pairs (NetworKIN score ≥1.5) were included in the analysis, and with this approach we identified 28 different kinases that had an inferred exercise-induced alteration in activity at a *q*-value of ≤ 0.05 (Supplementary Table 5). Notably, several of the identified kinases (e.g., MAPK1/3 and MAPKAPK2/3/5) presented with a cluster 2-like pattern of inferred activity which suggested that they might be part of the signaling pathway that shows prolonged activation specifically after a bout of resistance exercise (Fig. 3a).

Inspired by the outcomes from KSEAapp, we conducted a comparative analysis with the phosphopeptide data that was recently published by Blazev et al. (2022) ^14^. Specifically, Blazev et al. performed phosphoproteomic analyses on human vastus lateralis muscles at 0 and 3 hr after they had completed a bout of endurance or resistance exercise. Despite the obvious similarities, there were a number of important differences from our study including the use of a cross-over experimental design, bilateral exercises, different exercise movements, intensities and durations, and a vastly different phosphoproteomic workflow. Nonetheless, we reasoned that if a signaling pathway that shows prolonged activation specifically after a bout of resistance exercise truly exists, then our bioinformatic methods should be able to detect it in both studies. Hence, we subjected the original phosphopeptide data from Blazev et al. to the same processing procedures that were utilized for the analysis of our phosphopeptide data. The resulting dataset (Supplementary Table 6), in conjunction with KSEAapp, was then used to infer how the different modes of exercise affected the activity of the skeletal muscle kinome. In total, 37 kinases with an inferred exercise-induced alteration in activity at a *q*-value of ≤ 0.05 were identified (Supplementary Table 7). We then generated heatmaps to compare the Z-scores for all of the kinases that received inferences in both datasets, and from this comparison, it was clear that both datasets had led to very similar predictions (Fig. 3a,b). Indeed, as illustrated in Fig. 3c, we identified 14 kinases whose activity was inferred to be significantly affected by exercise in both datasets, and we also found that the cluster 2-like pattern for kinases such as MAPK1/3 and MAPKAPK2/3/5 was readily apparent in both datasets.

In KSEAapp, the Z-score for the inferred change in the activity of a given kinase is based on the change in the phosphorylation of its known and/or predicted substrates, as well as the number of substrates that it is linked with. Thus, to develop a better sense of factors that were driving the Z-scores for the kinases in Fig. 3c, we generated heatmaps that displayed the mean exercise-induced change in the phosphorylation of the known and/or predicted substrates of each kinase along with the number of substrates (m) that the Z-scores were derived from (Fig. 3d). In the heatmaps, we also listed the number of substrates that were common to both datasets $\left(m^{=}\right)$, as well as the combined number of distinct substrates $\left(m^{c}\right)$, and with this information we made two important observations. First, when comparing the two studies, we found that the exercise-induced changes in the phosphorylation of the substrates for each kinase were extremely similar and these results were obtained in the face of a low degree of overlap $(m^{=}/m^{c})$ between the substrates. In other words, the two independent studies produced very similar predictions about kinase activity, yet these predictions were derived from substantially different substrates (e.g., the average overlap in substrates for the kinases in Fig. 3c was only 32%).

Another important conclusion that could be drawn from Fig. 3c is that the large Z-scores that were observed for kinases such as MAPK1/3 were due to small increases in the phosphorylation of a high number of substrates which suggests that exercise did not lead to a robust increase in the activity of these kinases. On the other hand, the large Z-scores for kinases such as MAPKAPK2/3/5 were due to large increases in the phosphorylation of a low number of substrates (i.e., were presumably due to a robust increase in kinase activity). It also bears noting that the number of substrates that are linked to each of the kinases does not necessarily reflect the number of substrates that the kinases regulate in biological systems. Instead, the number of substrates reflects how well-studied the kinases are, and this can impact both the number of known substrates as well as the number of predicted substrates. For instance, MAPK1/3 are highly studied kinases and have 1,502 known substrates listed in PhosphoSitePlus, whereas MAPKAPK2/3/5 only have 122.

At this point, the outcomes from the two studies suggested that prolonged activation of signaling through MAPKAPK2/3/5 is part of a resistance exercise-specific signaling pathway. However, the number of substrates that led to this prediction was not high. Therefore, to increase the number of substrates that could be linked to MAPKAPK2/3/5 we took advantage of the kinase-substrate prediction module in PhosR which uses a machine learning method to predict the top three substrates for all kinases in the dataset that have a known substrate in its reference database ^8^. Fortunately, MAPKAPK2 was one of those kinases and, assuringly, one of the top three substrates predicted by PhosR was HSPB1(S15), a phosphorylation site that was recently shown to be regulated by MAPKAPK2 *in vivo* (Extended Figure 2) ^15^. The other top three substrates included CRYAB(S59) and XIRP1(S529), and as illustrated in Extended Figure 3, all three substrates presented with a cluster 2-like pattern in phosphorylation. Indeed, a cluster 2-like pattern of phosphorylation was observed for all of the known and/or predicted MAPKAPK2/3/5 substrates that were identified in both studies, and a side-by-side comparison of the two studies revealed that the magnitude of change in the phosphorylation for each substrate was remarkably similar (Extended Fig. 3). At this point it also bears mentioning that some of the MAPKAPK2 substrates listed in Extended Figure 3 have already been implicated in the regulation of skeletal muscle growth. For instance, our lab recently identified TRIM28(S473) phosphorylation as a potential regulator of skeletal muscle size and function ^7^, and changes in TSC2(S1254) phosphorylation have been linked to the regulation of mTORC1 (a kinase that is considered to play a key role in the regulation of protein synthesis and muscle mass) ^16,17^. Hence, there were ample reasons to suspect that the prolonged activation of MAPKAPK2/3/5 might contribute to the increase in protein synthesis and growth that occurs after a bout of resistance exercise.

### Prediction and validation of a signaling pathway that is activated specifically by resistance exercise.

**Figure 4.**
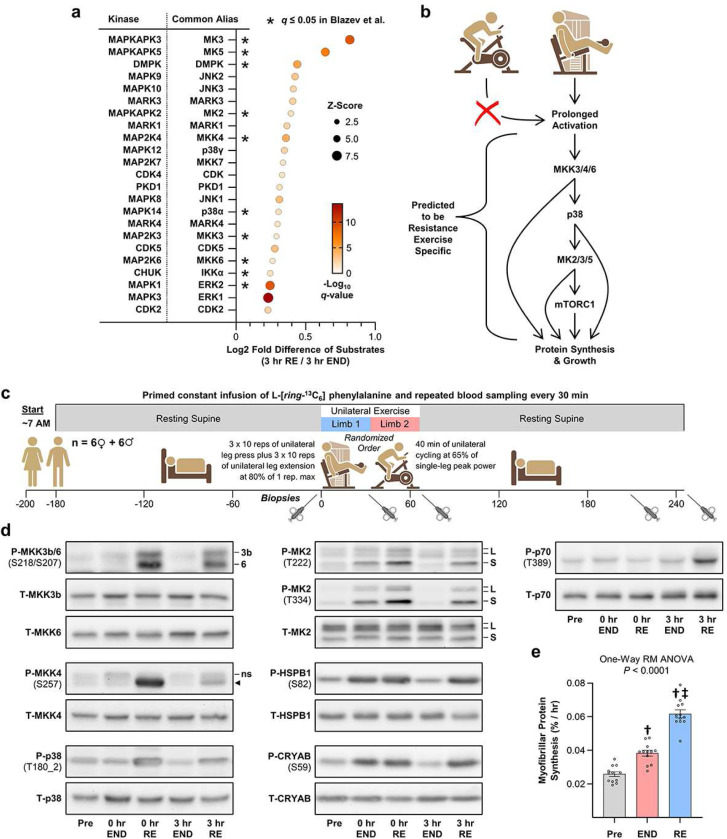
Prediction and validation of a signaling pathway that is activated specifically by resistance exercise in humans **a**, Multivariable plot with Z-scores, *q*-values, and the mean differences in the phosphorylation of the known and/or predicted substrates for each kinase that was inferred by KSEAapp to have a significant difference (*q* ≤ 0.05) in activity at 3 hr post resistance (3 hr RE) vs. 3 hr post endurance (3 hr END) exercise. The list includes the gene name of each kinase as well as its common alias. Also highlighted with a * are kinases that were inferred by KSEAapp to have significant differences in activity at 3 hr RE vs. 3hr END as determined from the phosphopeptides dataset published by Blazev et al., 2022 ^14^. **b**, Manually curated prediction of the interconnectivity and functional outcomes of the most robustly perturbed kinases listed in (**a**). **c**, Schematic of the experimental intervention that was used to test the predictions in (**b**). **d**, The samples from (**c**) were subjected to western blot analysis for the phospho (P) and total (T) levels of the indicated proteins. Non-specific band (ns), long isoform of MK2 (L), short isoform of MK2 (S). The quantitative analysis of the western blots are provided in Extended Figure 4. **e**, Measurement of the mean rate of myofibrillar protein synthesis during three different 3 hr windows of time including: i) 3 hr pre-exercise (Pre), ii) immediately post (0 hr) to 3 hr post END, and iii) 0 hr to 3 hr post RE. Values are group means ± SEM, n = 12 per group. Data was analyzed with one-way repeated measures (RM) ANOVA. † Significantly different from Pre, ‡ significantly different from END, *P* < 0.0001.

Next, we set out to identify the components of the resistance exercise-specific signaling pathway that drives the putative activation of MAPKAPK2/3/5. However, before doing this, it was important to consider that our previous KSEAapp inferences were based on comparisons with the pre-exercise state. This consideration was important because endurance and resistance exercise can induce the release of factors that have the potential to exert systemic alterations in kinase activity (e.g., exerkines) ^18^, and if such factors were released, then they could obscure our ability to resolve how endurance and resistance exercise differently affect the kinome. Fortunately, in our unilateral exercise model, the muscles subjected to each mode of exercise were exposed to the same systemic factors. Thus, to more clearly infer how by the different modes of exercise exerted prolonged effects on the activity of the kinome, the phosphorylation state of each substrate in the 3 hr post resistance exercise samples was expressed relative to what was observed in the 3 hr post endurance exercise samples. The resulting values were then used to make KSEAapp-based inferences of kinase activity (Supplementary Table 8), and all kinases with an inferred increase in activity at a *q*-value of ≤ 0.05 were displayed on a multivariable plot (Fig. 4a). To bolster the predictions, the same procedure was also performed with the phosphopeptide dataset from Blazev et al. (Supplementary Table 8), and the kinases that had an inferred increase in activity (*q*-value ≤ 0.05) were highlighted in Figure 4a. When combined, the results from these analyses identified several kinases that had a reproducibly inferred increase in activity, and we considered these kinases to be the most likely members of the signaling pathway that shows prolonged activation specifically after a bout of resistance exercise.

STRING ^19^ and literature-based searches were then used to develop a better understanding of the potential interconnectivity and functions of the kinases in Figure 4a that had a reproducibly inferred increase in activity. Notably, during the literature-based searches, the most abundant information was retrieved when the common alias, rather than the gene name, was used for each kinase. Accordingly, the common alias for each kinase was listed in Figure 4a, and the remainder of the manuscript will refer to kinases by their common alias rather than their gene name.

After performing our searches, the potential interconnectivity between many of the kinases had become readily apparent. For instance, previous studies have established that MKK3/4/6 can function as upstream regulators of p38 which, in turn, can function as an upstream regulator of MK2/3/5 (a.k.a. MAPKAPK2/3/5) ^20–22^. Moreover, numerous studies have implicated these kinases in the regulation of mTORC1, protein synthesis, and growth ^23–25^. As such, we hypothesized that prolonged activation of a signaling pathway involving MKK3/4/6, p38, MK2/3/5, and mTORC1 occurs specifically in response to resistance exercise and that the activation of this pathway would be sufficient to induce an increase in protein synthesis and skeletal muscle growth (Fig. 4b).

To test the validity of our hypothesis, we conducted a follow-up study with the same experimental design that was used in our phosphoproteomic analysis (Fig. 4c). However, in this case, the number of participants was increased (n = 12) and both men and women were included (age, 20.8 ± 0.6 years; BMI, 25.3 ± 1.2 kg/m^2^; VO_2_ peak, 45.4 ± 1.9 mL/kg/min, Supplementary Table 9). Moreover, after obtaining measurements of myofibrillar protein synthesis, the remaining portion of the biopsies were subjected to targeted analysis of specific phosphorylation events rather than global phosphoproteomic analyses (Fig. 4d,e). Specifically, we used western blot analysis to examine the phosphorylation of key regulatory residues on, and downstream substrates of, the kinases listed in our model (Fig. 4b). For instance, the S218, S257, and S207 residues are members of the activation loop of MKK3b (the dominant MKK3 isoform in skeletal muscle), MKK4, and MKK6, respectively ^20,26^, and all three of these residues showed a prolonged increase in phosphorylation specifically after resistance exercise (Fig. 4d and Extended Fig. 4a-c). Likewise, MKK3b, MKK4, and MKK6 can phosphorylate the T180 2 residues in the activation loop of p38, and these residues also showed a prolonged increase in phosphorylation specifically after the bout of resistance exercise (Fig. 4d and Extended Fig. 4d) ^20^. Upon activation, p38 can phosphorylate the T222 residue in the activation loop of MK2, as well as the T334 residue, which together support maximal activation of MK2 ^27^. Once again, both sites showed a prolonged increase in phosphorylation specifically after resistance exercise and this effect was present in both the long and short isoforms of MK2 (Fig. 4d and Extended Fig. 4e-h) ^27^. Moreover, we observed a prolonged increase in the phosphorylation of a known MK2 substrate (HSPB1(S82)), a predicted MK2 substrate (CRYAB(S59)), and the mTORC1 substrate p70(T389) (Fig. 4d and Extended Fig. 4i-k) ^28^. Hence, when taken together, the follow-up study provided consistent support for the notion that prolonged activation of a signaling pathway involving MKK3b/4/6, p38, MK2/3/5, and mTORC1 occurs specifically in response to resistance exercise.

### Identification of signaling events that correlate with the exercise-induced changes in myofibrillar protein synthesis.

**Figure 5.**
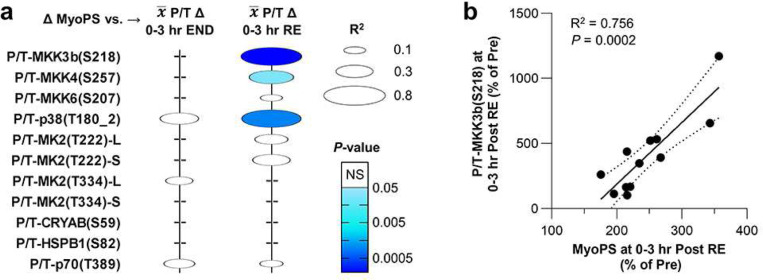
Changes in MKK3b(S218) phosphorylation are highly correlated with the resistance exercise-induced increase in myofibrillar protein synthesis For each of the signaling events analyzed in Figure 4, linear regression was used to compare each participant’s endurance (END) and resistance exercise (RE) induced change (Δ) in myofibrillar protein synthesis (MyoPS) with the mean $(\bar{x})\;\text{Δ}$ of the phospho to total protein ratio (P/T) at 0 hr and 3 hr post-exercise. **a**, Multivariable plot illustrating the coefficient of determination (R-squared) and *P*-value of the co-relationship for all comparisons that revealed an R-squared value of >0.1. NS indicates not significant. **b**, Graph of the co-relationship between the Δ in MyoPS and the Δ in the P/T for MKK3(S218). Individual values for each participant were expressed as a percentage of the value obtained in their respective pre-exercise sample. Dashed lines represent the 95% confidence intervals.

According to the model presented in Figure 4b, the magnitude of activation through the resistance exercise-specific pathway should correlate with the magnitude of the increase in myofibrillar protein synthesis, and the strongest correlations should be linked to the most upstream members of the pathway (i.e., MKK3b/4/6). To test this, linear regression was used to assess the relationships between the change in myofibrillar protein synthesis and the mean change in phosphorylation for each signaling event that was studied during the 3 hr post-exercise window. As shown in Figure 5, the outcomes revealed that the resistance exercise-induced increase in MKK3b(S218) phosphorylation and the increase in myofibrillar protein synthesis were very highly correlated events. Significant correlations were also observed with the resistance exercise-induced increase in MKK4(S257) and p38(T180_2) phosphorylation. However, the *P*-values for these correlations were not as strong, and unlike MKK3b(S218) phosphorylation, the correlations did not remain statistically significant when the change in phosphorylation at each of the individual post-exercise time points was considered (Extended Fig. 5). Of note, while the correlations in Figure 5 do not demonstrate causation, they are worthy of highlighting because i) they strongly align with the expectations of our model, and ii) they are dramatically stronger than previously reported predictors of a resistance exercise-induced increase in myofibrillar protein synthesis. Indeed, markers of mTORC1 activation such as p70(T389) phosphorylation are currently the most well-recognized predictors: however, previous studies have shown that these markers only produce R^2^-values of ~0.13 which pales in comparison to the R^2^-value (0.76) that was observed for MKK3b(S218) ^29,30^.

### Establishment of mouse models for studying the distinct effects of endurance and resistance exercise.

**Figure 6.**
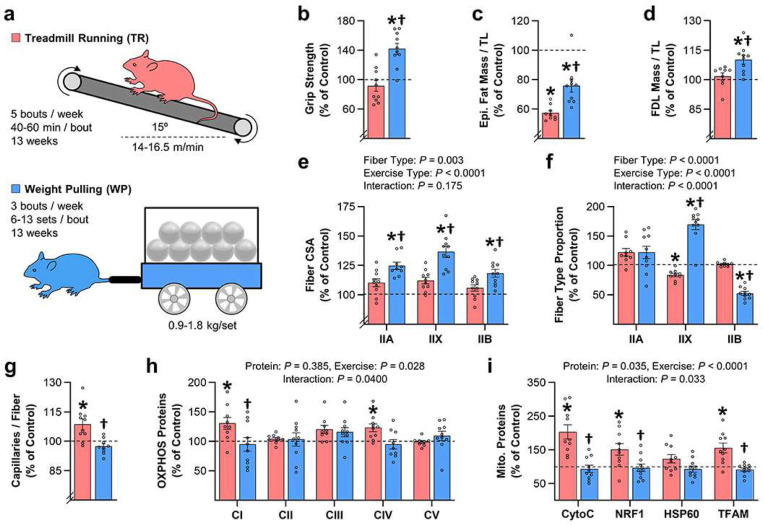
Mouse models of endurance and resistance exercise lead to distinct adaptations **a**, Mice were subjected to 13 weeks of endurance exercise with treadmill running (TR) or resistance exercise with weight pulling (WP). Values in each exercise group were expressed relative to their respective mock-trained (control) groups (see Extended Figures 6, 7 and Zhu et al. 2021 ^33^), and then the effects of the different modes of exercise were compared. **b**, Measurements of grip strength. **c-d**, The mass of the individual epididymal (Epi.) fat pads (**c**), and flexor digitorum longus (FDL) muscles (**d**), after being normalized to tibia length (TL). **e-g**, The average cross-sectional area (CSA) of the different fiber types (**e**), the proportion of the fibers that were represented by each fiber type (**f**), and the average number of capillaries per fiber (**g**), as assessed in mid-belly cross-sections from FDL muscles. **h-i**, FDL muscles were subjected to western blot analysis for (**h**) members of the five OXPHOS complexes (i.e., CI - CV), and (**i**) other mitochondrial (mito.) proteins. Values in the graphs are presented as the group means ± SEM, n = 8–10 per group. * Significantly different from the condition-matched control group as presented in Extended Figures 6, 7 and Zhu et al. 2021, or † significant difference between the effect of endurance and resistance exercise, *P* < 0.05. The data were analyzed with Student’s t-tests (**b-d**, and **g**), or two-way ANOVA (**e, f, h**, and **i**).

Previous studies have demonstrated that the distinct adaptations that occur in response to endurance and resistance exercise in humans are conserved in lower organisms such as mice ^31,32^. Hence, we reasoned that if prolonged activation of signaling through MKK3b/4/6, p38, MK2/3/5, and mTORC1 actually drives the growth-promoting effect of resistance exercise, then the resistance exercise-specific activation of this pathway should also be conserved. Therefore, to test this, we utilized our recently described mouse model of resistance exercise called weight pulling (WP) ^33^, along with treadmill running (TR) as a model for endurance exercise. Importantly, our initial work with these models revealed that long-term training with TR did not induce changes in grip strength, the mass of skeletal muscles such as the flexor digitorum longus (FDL), or the cross-sectional area of the fibers in the FDL, but it did lead to significant increases in markers of aerobic capacity such as capillary density and the content of various mitochondrial proteins (Fig. 6 and Extended Fig. 6). In stark contrast, long-term training with WP induced significant increases in grip strength, the mass of the FDL, and the cross-sectional area of the fibers in the FDL, but it did not induce alterations in markers of aerobic capacity (Fig. 6 and Extended Fig. 7).

The aforementioned outcomes indicated that our mouse models are able to recapitulate the distinct adaptations that occur when humans engage in endurance versus resistance exercise ^1,2^. Thus, having established this point, we then used these models to determine whether the resistance exercise-specific activation of signaling through MKK3b/4/6, p38, MK2, and mTOR is a conserved phenomenon. For instance, in our first experiment, the workflow shown in Extended Figure 8 was used to identify the signaling responses that occur immediately (0 hr) after a bout of TR or WP, and the outcomes revealed that WP induced robust activation of signaling through all of the markers of the putative resistance exercise-specific pathway. In constrst, TR had very little, if any, effect on the same markers (Extended Fig. 8).

**Figure 7.**
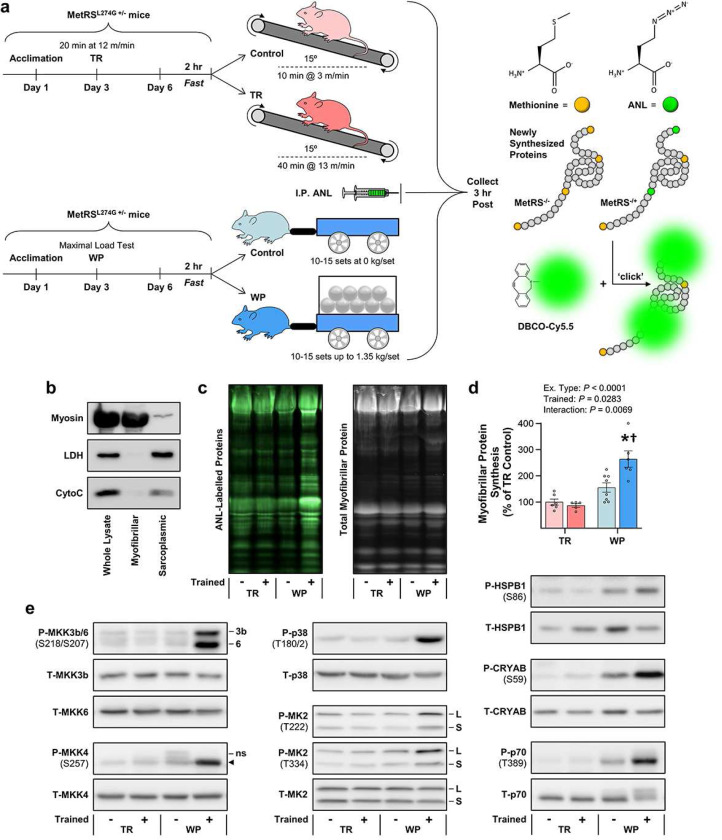
Mouse models affirm that prolonged activation of signaling through MKK3/4/6, p38, MK2, and mTORC1 occurs specifically in response to resistance exercise **a**, Schematic of how transgenic mice that express a mutated form of the methionyl-tRNA synthetase (MetRS^L274G +/−^) were subjected to endurance exercise with treadmill running (TR), resistance exercise with weight pulling (WP), or their respective mock-trained (control) conditions. At the end of the last training bout, the mice were injected with azidonorleucine (ANL) which is an azide-bearing analog of methionine that can be incorporated into newly synthesized proteins of mice that possess the MetRS^L274G^ transgene. The FDL muscles were collected at 3 hr post-training and subjected to the analyses described below. **b**, The muscles were homogenized and whole lysates were separated into myofibrillar and sarcoplasmic fractions. The different fractions were then subjected to western blot analysis for myofibrillar and sarcoplasmic proteins. **c**, As illustrated in (**a**), a DBCO-based ‘click’ reaction was used to label the ANL-containing proteins with a fluorophore (Cy5.5). Fluorescently labeled proteins in the myofibrillar fraction were then subjected to SDS-PAGE and used to visualize the in-gel amount of ANL-labeled proteins as well as the total amount of protein in each sample. **d**, The ANL-labeled to total protein ratio for each sample was quantified and used as a readout for the rate of myofibrillar protein synthesis. **e**, The FDL muscles were subjected to western blot analysis for the phospho (P) and total (T) levels of the indicated proteins. Non-specific band (ns), long isoform of MK2 (L), short isoform of MK2 (S). The quantitative analysis of these western blots is provided in Extended Figure 9. Values in the graph are presented as the group mean ± SEM, n = 4–8 per group. Data were analyzed with two-way ANOVA. * Significantly different from TR control, or † WP control, *P* < 0.05.

After obtaining the above results, we performed a second experiment in which we focused on the prolonged signaling responses (Fig. 7a). In this experiment, we also wanted to know whether TR and WP had differential effects on the induction of myofibrillar protein synthesis. Thus, to obtain such measurements, we employed mice that express a mutated form of the methionyl-tRNA synthetase (MetRS^L274G +/−^) ^34^. As illustrated in Figure 7b-c, these mice were important because they allowed us to develop a novel in-gel method for visualizing and quantifying the amount of newly synthesized proteins that accumulated in the myofibrillar fraction during the 3 hr post-exercise window (i.e., the rate of myofibrillar protein synthesis). As expected, the results with these mice revealed that WP led to a significant increase in the rate of myofibrillar protein synthesis whereas TR had no effect (Fig. 7c,d). Furthermore, it was determined that WP led to prolonged activation of signaling through all of the markers of the putative resistance exercise-specific pathway whereas, again, TR had no effect (Fig. 7e and Extended Fig. 9). Hence, it can be concluded that the prolonged activation of signaling through MKK3b/4/6, p38, MK2, and mTORC1 that occurs specifically in response to resistance exercise in humans is a conserved phenomenon.

### Prolonged activation of MKK3b and MKK6 is sufficient to induce the resistance exercise-specific signaling events, protein synthesis, and growth.

**Figure 8.**
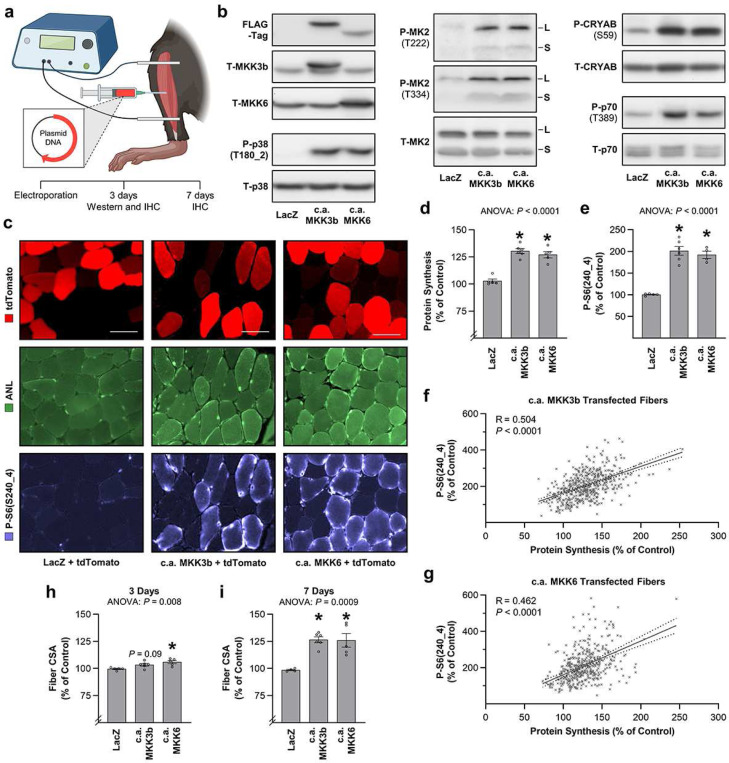
Genetic activation of MKK3b and MKK6 is sufficient to induce resistance exercise-specific signaling events, protein synthesis, and growth **a**, Schematic describing the electroporation procedure that was used to transfect mouse tibialis anterior (TA) muscles. **b**, TA muscles of C57BL6 mice were transfected with plasmid DNA encoding FLAG-tagged and constitutively active (c.a.) mutants of human MKK3b or MKK6, or with LacZ as a control condition. The muscles were collected at 3 days post-transfection and subjected to western blot analysis for the phospho (P) and total (T) levels of the indicated proteins. Long isoform of MK2 (L), short isoform of MK2 (S). Quantitative analysis of the western blots is provided in Extended Figure 10. **c**, TA muscles of MetRS ^+/−^ mice were co-transfected with tdTomato and c.a. MKK3b, c.a. MKK6, or LacZ as the control condition. At 3 days post-transfection the mice were injected with ANL to label newly synthesized proteins. At 3 hr after the ANL injection, the muscles were collected for histological analysis. Cross-sections were used to visualize the transfected (tdTomato positive) vs. non-transfected (control) fibers, ANL-labeled proteins, and phosphorylated (P) S6(S240_4) as a marker of signaling through mTORC1, scale bars = 50 μm. For each sample, the intensity of the signal for ANL (i.e., protein synthesis) (**d**), and P-S6(S240_4) (**e**) were simultaneously measured in randomly selected fibers, and then the values in the transfected fibers were expressed relative to the mean values observed in the non-transfected fibers within that sample (n = 60–120 transfected and non-transfected fibers per sample). **f,g**, Graphs of the co-relationships between protein synthesis and P-S6(S240_4) in the fibers from (**d,e**) that were transfected with c.a. MMK3b (**f**), or c.a. MKK6 (**g**). The data were analyzed with linear regression, dashed lines represent the 95% confidence intervals. **h**, The cross-sectional area (CSA) of the same fibers analyzed in (**d,e**) was measured and then the CSA of the transfected fibers was expressed relative to the mean of the non-transfected fibers within that sample. **i**, TA muscles of C57BL6 mice were transfected, collected at 7 days post-transfection, and analyzed for fiber CSA as described above (n = 46–140 transfected and non-transfected fibers per sample). Values in the graphs are presented as the group mean ± SEM, n = 4–5 samples per group (290–526 fibers per group). The data in (**d,e,h,i**) were analyzed with one-way ANOVA. * Significantly different from LacZ, *P* < 0.05.

According to our model (Fig. 4b), the prolonged activation of MKK3b, MKK4, and/or MKK6 that occurs after a bout of resistance exercise should be sufficient to induce signaling through the downstream members of the putative resistance exercise-specific pathway (e.g., p38, MK2, and mTORC1). Hence, to test this, gain-of-function experiments were performed in mouse tibialis anterior (TA) muscles. Specifically, electroporation was used to transfect TA muscles with plasmid DNA encoding FLAG-tagged and constitutively active (c.a.) mutants of human MKK3b, MKK4 or MKK6, or LacZ as a control condition (Fig. 8a). Unexpectedly, at 3 days post-transfection, we found that the plasmid DNA encoding c.a. human MKK4 was not well expressed, and a similar, but less severe, limitation was encountered when plasmid DNA encoding a GST-tagged c.a. mutant of mouse MKK4 was employed (Supplemental Figure 1). Of greater importance, neither variant of c.a. MKK4 induced an increase in the phosphorylation of its canonical substrates (e.g., p38 and JNK), and this precluded us from performing additional gain-of-function experiments with MKK4 (Supplemental Fig. 1) ^35^. On the other hand, both c.a. MKK3b and c.a. MKK6 were readily expressed, and as shown in Figure 8b and Extended Figure 10, both led to a robust increase in signaling through the downstream members of the putative resistance exercise-specific pathway.

Following up on these observations, another prediction from our model is that the prolonged activation of MKK3b and/or MKK6 should be sufficient to induce an increase in protein synthesis. To assess this prediction, our MetRS^L274G +/−^ mice were used to develop a novel histological method for visualizing and quantifying the amount of newly synthesized proteins that accumulate at the single fiber level. Namely, TA muscles were co-transfected with plasmid DNA encoding c.a. MKK3b, c.a. MKK6, or LacZ, along with tdTomato as a reporter for the transfected versus non-transfected fibers. Newly synthesized proteins were labeled by injecting the mice with ANL 3 hr prior to collection, and the collections were performed with a perfusion fixation method that included the use of phosphatase inhibitors in the perfusate. Importantly, by using this approach, we were able to simultaneously quantify the amount of newly synthesized and the phosphorylation state of different signaling molecules at the single fiber level. For instance, within the same fibers, we quantified both the amount of S6 that was phosphorylated on the S240_4 residues (a commonly used marker of mTORC1 signaling) ^25^, as well as the amount of ANL-labeled proteins, and the outcomes revealed that the prolonged activation of MKK3b and MKK6 was sufficient to induce an increase in both S6 phosphorylation and the amount of ANL-labeled proteins (i.e., protein synthesis) (Fig. 8c-e). Reminiscent of the results obtained in humans after resistance exercise, the single fiber level data also revealed that the magnitude of activation of MKK3b- and MKK6-induced signaling was highly correlated with the magnitude of the increase in protein synthesis (Fig. 8f,g).

Inspired by the above outcomes we proceeded to assess the final, and perhaps the most notable, part of our model which predicts that the prolonged activation of MKK3b and/or MKK6 would be sufficient to induce skeletal muscle growth. We first tested this by measuring the cross-sectional area (CSA) of the same transfected and non-transfected fibers that were analyzed in Figure 8c-g. As a reminder, these were from muscles that were only at 3 days post electroporation, yet, even after this short amount of time, c.a. MKK6 had led to a significant increase in fiber CSA, and a similar trend (*P* = 0.09) was observed for c.a. MKK3b (Fig. 8h). More importantly, when we analyzed muscles at 7 days post electroporation, it was clear that both c.a. MKK3b and c.a. MKK6 had led to a robust increase in fiber size (Fig. 8i).

## Discussion

The initial goal of this study was to identify phosphorylation events that are differentially regulated by endurance versus resistance exercise. After achieving this goal, we focused on a cluster of events that presented with a prolonged elevation in phosphorylation specifically after resistance exercise. Within this cluster, we found a prominent over-representation of gene ontology terms such as “translation” and the “positive regulation of skeletal muscle growth,” and our bioinformatics-based analyses indicated that the cluster could be linked to prolonged activation of a signaling pathway that involved MKK3b/6, p38, MK2, and mTORC1. Follow-up studies in both humans and mice confirmed that prolonged activation of signaling through these kinases occurs specifically after resistance exercise. Moreover, gain-of-function analyses demonstrated that prolonged activation of MKK3b and MKK6 are sufficient to induce signaling through p38, MK2, and mTORC1, as well as promote an increase in protein synthesis and skeletal muscle growth. Given what is known about the interconnectivity of these kinases, we propose that the pathway illustrated in Figure 4b represents the basic framework of a signaling cascade that plays a major role in the growth-promoting effects of resistance exercise.

While the outcomes of this study offer many insights into the potential mechanisms that drive exercise mode-specific adaptations, they also raise several new questions. For example, in the future, it will be important to determine whether MKK3b and/or MKK6 are necessary for resistance exercise-induced activation of p38, MK2, mTORC1, protein synthesis, and growth. Although the significance of this question is easy to recognize, answering it will not be a trivial task. Specifically, the double knockout of MKK3 and MKK6 is embryonic lethal and, therefore, the development of skeletal muscle-specific and inducible double knockout mice will likely be needed ^36^. Following up on this point, it is also important to consider that MKK3b and MKK6 share ~80% amino acid sequence homology, and both are well recognized for their abilities to activate the various isoforms of p38 ^20^. Indeed, consistent with the outcomes from our gain-of-function analyses, it has been shown that MKK3b and MKK6 often exert redundant effects ^36,37^. However, there are also many instances in which MKK3 and MKK6 have been shown to exert distinct regulatory functions ^37^. The later point is particularly noteworthy because, as shown in Figure 5, we found that the increase in activation loop phosphorylation on MKK3b following resistance exercise was very highly correlated with the induction of myofibrillar protein synthesis (R^2^ = 0.76, *P* = 0.0002); whereas no such effect was observed for MKK6 (R^2^ = 0.10, *P* = 0.37). Hence, we suspect that future studies will reveal that MKK3b alone, rather than MKK3b and/or MKK6, is essential for the growth-promoting effects of resistance exercise.

Our discovery that prolonged activation of MKK3b and MKK6 is sufficient to induce skeletal muscle growth also raises questions about the downstream mechanisms via which these kinases exert their impacts. When contemplating the possibilities, one of the most obvious candidates involves the activation of signaling through mTORC1. Namely, our results indicate that prolonged activation of MKK3b and MKK6 induced signaling through mTORC1, and previous work from our lab has shown that the activation of mTORC1 is sufficient to induce an increase in protein synthesis and skeletal muscle growth ^38^. Hence, it is likely that the increase in protein synthesis and growth was at least partially mediated by the activation of mTORC1. Importantly, however, a variety of mTORC1-independent mechanisms could also be involved. For instance, several lines of evidence indicated that the prolonged activation of MKK3b and MKK6 induced signaling through p38 and MK2, and previous studies have shown that the activation of these kinases can exert mTORC1-independent effects on protein synthesis. As a case in point, MK2 can directly phosphorylate the S15 and S82 residues on HSPB1, and the phosphorylation of these residues has been linked to enhanced formation of the eIF4F translation initiation complex ^39,40^. Moreover, p38 can directly phosphorylate and activate the MAPK-interacting kinases as well as the eukaryotic elongation factor 2 kinase which, in turn, are known to regulate translation initiation and elongation via phosphorylation of the S209 and T56 residues on eIF4E and eEF2, respectively ^25^. Lastly, recent studies have also suggested that MKK3 might function as a hub that controls much more than just p38-dependent events ^41,42^. For example, MKK3 can directly interact with and induce the transcriptional activity of MYC, a transcription factor that has been widely implicated in the induction of ribosome biogenesis and growth ^42,43^. Simply put, there are numerous ways in which the activation of MKK3b and MKK6 could induce protein synthesis/growth, and we expect that a deeper understanding of these mechanisms will shed light on the fundamental processes via which resistance exercise leads to an increase in muscle mass.

A final question that is worthy of discussion relates to how resistance exercise induces prolonged activation of signaling through MKK3b and MKK6. On this note, it is important to consider that MKK3b and MKK6 function as part of the neck in an hourglass-shaped signaling cascade whereby a multitude of inputs and outputs are mediated through a small set of common intermediates (e.g., MKK3b and MKK6) ^44^. In terms of inputs, the canonical activators of MKK3b and MKK6 belong to a large and diverse group of kinases called MAP3Ks. Specifically, the human genome encodes at least 24 different MAP3Ks and these kinases are capable of responding to a plethora of stimuli, including mechanical perturbations ^45^. For example, previous studies have shown that mechanical stimuli can promote signaling through at least two MAP3Ks that are expressed in skeletal muscle (ZAKβ and TAK1) ^46,47^. Intriguingly, these same studies have shown that ZAKβ plays a major role in the activation of signaling through p38 that occurs immediately after a bout of electrically evoked contractions ^46^ and that TAK1 is necessary for the increase in fiber size that occurs after two weeks of chronic mechanical overload ^47^. Moreover, recent work by Roy and Kumar (2022) demonstrated that prolonged activation of TAK1 is sufficient to induce an increase in protein synthesis and skeletal muscle growth ^48^. Thus, a seemingly fruitful topic for future studies will be to determine whether there are any MAP3Ks that are activated specifically by resistance exercise, as the identification of such kinases would not only offer insight into the mechanisms via which skeletal muscles sense and respond to the stimuli that are specific to resistance exercise (e.g., resistance exercise-specific mechanosensors), but they could also serve as excellent targets for drugs that are aimed at mimicking the growth-promoting effects of resistance exercise.

In summary, our study has identified a diverse array of phosphorylation events that are differentially regulated by endurance versus resistance exercise, and with this information, we were able to discover the basic framework of a signaling pathway that is activated specifically by resistance exercise. Moreover, our novel method for visualizing and quantifying newly synthesized proteins enabled us to demonstrate that activation of this pathway is sufficient to induce an increase in protein synthesis and skeletal muscle growth. In the future, we believe that it will be particularly important to identify the upstream regulators of this pathway and to determine whether the pathway can serve as an effective target for therapies that strive to restore the loss of muscle mass that occurs during conditions such as disuse, cancer cachexia, and aging.

## Methods

### Exercise Protocol in Human Participants

The procedures for Cohort 1 (phosphoproteomics study) and Cohort 2 (follow-up study) were identical. In total, six females and ten males were recruited to participate. The participants were healthy, between the ages of 19 and 26, and had experience in aerobic and resistance exercise but were not regularly training (Supplementary Tables 1 and 9). The study (https://clinicaltrials.gov/study/NCT04263714) was approved by the Hamilton Integrated Research Ethics Board (HIREB #2196) and conformed to the standards for the use of human subjects in research as outlined by the Canadian Tri-Council Policy (TCPS 2 2022) on the ethical use of human subjects in research (https://ethics.gc.ca/eng/policy-politique_tcps2-eptc2_2022.html), as well as the declaration of Helsinki. Each participant was given written and oral information regarding the purpose of the study, experimental procedures, and potential risks before written informed consent was obtained.

#### Study Overview

In a within-subject repeated measures design, the participants visited the laboratories at the Department of Kinesiology on 6 separate occasions. On the first visit, participants were screened and informed consent was obtained. Participants were then assessed for body composition using dual-energy x-ray absorptiometry (DXA) after an overnight fast (~10 h), followed by acclimation with the resistance exercise and endurance exercise equipment. Participants’ legs were randomly assigned to the resistance or endurance exercise group (see “Exercise Protocols” below). On visits 2 and 4, participants completed 1-repetition maximum testing. On visits 3 and 5, participants completed a VO_2_ peak test on a cycle ergometer. These tests were used to determine the intensity of cycling and resistance exercise load on the day of the experimental trial (visit 6). For 3 days before visit 6, participants refrained from strenuous exercise and consumed a controlled diet (Supplier: Heart to Home meals) ^49^ to ensure minimum protein intake >1.2 g/kg with each participant’s energy requirement determined by the Harris-Benedict equation for basal metabolic rate and multiplied by an activity factor of 1.55, corresponding to light/moderately active individuals ^50^.

On visit 6, participants arrived at the laboratory at ~6:30 AM after an overnight fast. Catheters were placed in the antecubital vein on each arm: one for the venous blood sample and one for the infusion of the stable isotope L-[*ring*-^13^C_6_] phenylalanine. At ~7:00 AM, baseline blood samples were drawn, and then participants received a priming dose of the stable isotope (2 μmol/kg) before initiating a constant tracer infusion of 0.05 μmol/kg/min. Participants rested on a bed for the duration of visit 6 (excluding during the exercise protocols), and blood samples were taken every 20–30 min with evacuated heparinized tubes. After 180 min of rest, a muscle biopsy was taken from the vastus lateralis of a randomly selected leg. Upon completion of the biopsy, participants initiated the unilateral aerobic and resistance exercise protocols in a randomly assigned order (Supplementary Tables 1 and 9). Up to 10 min of rest was allotted between the two exercise protocols and within 5 min after the completion of each exercise protocol, a post-exercise (0 hr) biopsy was taken. After completing both exercise protocols, the participants rested on a bed while remaining in a fasted state, and additional biopsies were taken 3 hr after the completion of each exercise protocol.

#### Exercise Protocols

##### 1-repetition maximum (1RM) testing:

The participants engaged in a brief warm-up and then performed each unilateral exercise at approximately 50% of their estimated 1RM. As previously described, the load was then increased by 5–20% for each subsequent repetition until a true 1RM was achieved ^51^. Three to five min of rest was given between each attempt, and a successful attempt required the participant to move the load with the correct form throughout the full range of motion.

##### VO_2_ peak testing:

The participants completed double-leg and single-leg incremental peak oxygen uptake (VO_2_ peak) tests on a cycle ergometer (Velotron, RacerMate) as previously described ^52^. Briefly, a metabolic cart with an on-line gas collection system (Moxus modular oxygen uptake system, AEI Technologies) measured oxygen consumption (VO_2_) and carbon dioxide production (VCO_2_) data, and heart rate (HR) was monitored continuously with a HR monitor (Polar A3). The test began with a two-min warm-up at 50 watts (W), after which the power was increased by 1 W every two (bilateral) or four (unilateral) sec until volitional exhaustion or the point at which pedal cadence fell below 60 rpm. VO_2_ peak was defined as the highest oxygen consumption achieved over a 30 sec period. Single-leg peak power was the highest power output achieved during the test. The participant was deemed to have reached VO_2_ peak if their HR was within 5 beats per min of the age-predicted maximal HR; the respiratory exchange ratio was >1.2, and a plateau was reached in their oxygen consumption.

##### Visit 6 exercise protocols:

For the bout of resistance exercise, the participants used one leg to perform 3 sets of 10 reps of unilateral leg presses at 80% of their 1 RM followed by 3 sets of 10 reps of unilateral leg extensions at 80% of their 1RM, with 2 min of recovery allotted between the sets and exercises. For the bout of endurance exercise, the contralateral leg was used to perform 40 min of continuous unilateral cycling at 65% of the participant’s single-leg peak power.

#### Biopsies

All biopsies were taken after administration of 1% xylocaine local anesthesia with the use of a 5-mm Bergström needle that was adapted for manual suction. Muscle tissue samples were rapidly freed from any visible connective and adipose tissue, frozen in liquid nitrogen, and stored at −80°C for further analysis.

#### Myofibrillar Protein Synthesis

A portion of each biopsy (~30 – 50 mg) was homogenized using a TissueLyser (Qiagen) for two cycles, each lasting 40 sec at 20 Hz, in 10 μL/mg of ice-cold Buffer A (25 mM Tris-HCl pH 7.2, 0.5% Triton X-100, along with one PhosSTOP tablet (Roche) and one complete protease inhibitor tablet (Roche) added to 10 mL of buffer immediately before use). The homogenate was then centrifuged at 2,250g for 10 min at 4°C to separate the sarcoplasmic and myofibrillar protein fractions. The supernatant containing sarcoplasmic proteins was discarded, while the myofibrillar pellet was retained. The myofibrillar pellet was washed with doubly distilled water (500 μL), vortexed, and centrifuged at 250g for 10 min at 4°C. The supernatant was discarded, and the pellet was incubated in 0.3 M NaOH for 30 min at 50°C to solubilize the myofibrillar protein fraction. After vortexing, and centrifugation at 11,200g for 5 min at 4°C, the supernatant containing myofibrillar proteins was collected, and the pellet was discarded. This process was repeated, and the supernatants were pooled. Myofibrillar proteins were precipitated by adding 1 mL of 1M perchloric acid and centrifuging at 700g for 10 min at 4°C. The supernatant was discarded, and the protein-enriched pellets were washed twice with 70% ethanol. After removing ethanol, the pellets were treated with 1 mL 1M activated Dowex H+ (50XW8 Cation exchange resin) and 1 mL 1M HCL. The protein-enriched pellets were hydrolyzed at 110°C for 72 hr to release their respective amino acids. Following hydrolysis, the amino acids were purified by ion exchange chromatography on Dowex H+ resin using 2M NH_4_OH. After isolation and purification, the samples were dried under nitrogen, reconstituted in 0.1M HCl, and shipped to Metabolic Solutions^™^ for myofibrillar L-[*ring*-^13^C_6_] phenylalanine enrichment analysis.

Plasma L-[*ring*-^13^C_6_] phenylalanine enrichment was determined as previously described ^53^ and served as the precursor for calculating the rate of myofibrillar protein synthesis (MPS) using the precursor-product equation.


$$\text{MPS}=\left(\frac{\left[E_{2b}-E_{1b}\right]}{\left[E_{ic}\times t\right]}\right)\times 100$$

Here, $E_{\text{b}}$ represents the enrichment of bound myofibrillar protein, $E_{\text{ic}}$ is the average intracellular enrichment between two biopsies, and t is the tracer incorporation time in hours. Participants were ‘tracer naïve’, meaning they had not previously participated in a study involving L-[ring-^13^C_6_] phenylalanine infusion. Therefore, a pre-infusion blood sample was used to calculate resting myofibrillar MPS ^54^.

### Mass Spectrometry and Analysis

A portion of each biopsy was homogenized in 1 mL of Buffer B (40 mM Tris (pH 7.5), 1 mM EDTA, 5 mM EGTA, 0.5% Triton X-100, along with one PhosSTOP tablet (Roche) and one Complete Mini EDTA-Free Protease Inhibitor Cocktail Tablet (Roche) per 10 mL). Samples were homogenized using a Polytron (PT 1200 E) for 20 sec and then centrifuged at 6,000g for 1 min to remove bubbles and ensure complete homogenization. The homogenate was subsequently separated into pellet and supernatant fractions as previously described ^55^.

#### Protein digestion and peptide desalting

Proteins from each of the fractions were precipitated by adjusting the sample solution to 90% MeOH concentration by volume and centrifuging at 12,000g for 5 min. The supernatant was removed, and the protein precipitate was resuspended in 8 M urea, 50 mM Tris (pH 8.0), 10 mM TCEP, and 40 mM chloroacetamide, followed by a 30 min incubation with shaking to achieve complete reduction and alkylation of proteins. The sample was diluted to 1.5 M urea with 50 mM Tris (pH 8.0) and digested with trypsin (1:50 enzyme to protein ratio) at 37°C for 15 hr. The enzymatic digestion was quenched by acidifying the sample to pH < 2 with 10% trifluoroacetic acid (TFA). Strata-X desalting columns (Phenomenex) were conditioned by passing 1 mL of 100% acetonitrile (ACN) followed by 1 mL of 0.1% TFA through each column. Individual samples were centrifuged, and the acidic supernatant was collected and filtered through the Strata-X columns by gravity flow. The bound peptides were washed with 1 mL of 0.1% TFA and eluted into a fresh tube using 500 μL of 40% ACN and 0.1% TFA, followed by an additional elution with 300 μL of 80% ACN and 0.1% TFA. Eluted peptides were dried using vacuum centrifugation. Prior to TMT labeling, peptide concentrations were determined using a Pierce Quantitative Colorimetric Peptide Assay (Thermo Fisher Scientific).

#### TMT labeling

Two 10-plex and two 11-plex experiments were conducted as depicted in Figure 1a. In each experiment, 1 mg of peptides from each sample was incubated with one tandem mass tag (TMT) label from a kit (Thermo Fisher Scientific), following the manufacturer’s instructions. After incubation with shaking for 3 hr at room temperature, the reaction was quenched using 5% hydroxylamine and further incubated at room temperature for 15 min with shaking. An aliquot from each sample was combined in a 1:1 ratio across all channels and analyzed using an Orbitrap Elite mass spectrometer (Thermo Fisher Scientific) to confirm complete TMT peptide labeling and to compare peptide ratios in a “test mix”. These initial mixing ratios guided the creation of a final sample mix, where samples were combined at a 1:1 ratio. The resulting pooled sample containing TMT-labeled peptides from all samples was desalted using Strata-X desalting columns. Subsequently, the pooled sample was enriched with titanium dioxide immobilized on magnetic beads (MagReSyn, Resyn Biosciences). The remaining samples were preserved for proteome quantification. The enriched and unenriched samples were both separated via semipreparative, high pH reverse phase chromatography. The resulting fractions were dried using a vacuum centrifuge and reconstituted in MS-grade water with 0.2% formic acid for subsequent mass spectrometry analysis.

#### Nano-LC-MS/MS methods

Each sample was analyzed using an LTQ-OT-IT tribrid mass spectrometer (Orbitrap Fusion Lumos, Thermo Fisher Scientific) coupled with upfront nano-liquid chromatography (LC) separation. Samples underwent MS1 scans with an automatic gain control (AGC) target of 1×10^6^ ions and maximum injection times of 50 msec. MS1 scans were conducted at a resolving power of 60,000, with a scan range of 300 to 1500 m/z. Precursor ions with charge states ranging from +2 to +8 were selected for fragmentation and subsequent MS2 analysis. MS2 scans were performed with a 3 Th isolation window, high-energy collision dissociation (HCD) fragmentation at 35% normalized collision energy (NCE), and a dynamic exclusion duration of 30 sec. The resulting product ions were analyzed in the Orbitrap at a resolving power of 30,000, with an AGC target of 2×10^5^ ions and maximum injection times of 118 msec. Monoisotopic precursor selection and dynamic exclusion for 60 sec were enabled to enhance spectral quality and peptide identification. RAW data files are deposited in MassIVE (see Data Availability).

#### MS data analysis

The RAW data files were analyzed with COMPASS software suite against a target-decoy database of Homo sapiens proteins, downloaded from Uniprot on 09/16/2018 ^56^. Peptide and phosphopeptide datasets were searched with a 50-ppm precursor mass tolerance and a 0.02 Da fragment tolerance for b and y ions generated by HCD fragmentation. All fractions were searched with static carbamidomethylation of cysteine residues, static TMT 10-plex and 11-plex modifications of peptide N-termini and lysines, and dynamic methionine oxidation. Phosphopeptide fractions were also searched with additional dynamic phosphorylation modifications of serine, threonine, and tyrosine residues. Peptide identifications were filtered to a 1% false discovery rate (FDR). Peptides were then mapped back to their parent proteins and filtered to a 1% FDR at the protein level. Phosphorylation sites were considered localized if they received a localization score greater than 0.75 using the PhosphoRS algorithm embedded in COMPASS ^57^. TMT reporter ion signals were used for protein and phosphopeptide quantitation and were extracted from COMPASS result tables. Peptide and phosphopeptide intensities were first normalized to the total reporter ion intensity. Then, each TMT analysis was further normalized to an internal control sample (Subject B Pre-exercise condition), which allowed for quantitative comparisons between the individual TMT analyses. For the proteomics dataset, the samples from each subject were expressed relative to the pre-exercise condition, log2 transformed, and then subjected to post hoc analyses with moderated t-tests implemented in the LIMMA package in R (version 3.12.0) ^58^, and false discovery rate (FDR) correction applied using the Benjamini-Hochberg method ^59^ (Supplementary Table 2). The normalized phosphopeptide data set (referred to as “Pre PhosR”) can be found in Supplementary Table 3 and was subjected to further processing as described below.

#### Phosphoproteomic data processing and analysis

Modifications of the PhosR data processing package were used to mitigate the batch effects that were introduced by the different TMT analyses ^8^. Specifically, the package identified stably phosphorylated sites in the “Pre PhosR” dataset, and these were incorporated as ‘negative controls’ in a wrapper function (RUVphospho) to correct for batch effects. The parameter *k*, representing the number of experimental groups within the dataset, was set to 5 for the normalization of the phosphopeptide dataset that was generated in this study, and to 6 for the normalization of the phosphopeptide dataset that was published by Blazev et al. 2022 ^14^ (downloaded from https://github.com/JeffreyMolendijk/exercise_modalities/blob/main/data/input/human_phospho.xlsx, labeled as “Blazev - Pre PhosR” in Supplementary Table 6). Following batch effect correction, hierarchical clustering and principal component analysis were conducted using the ‘plotQC’ function in PhosR. The modified PhosR R programming code for the processing of the current and Blazev et al., datasets can be found in Supplementary Software 1 and 2, respectively.

The outputs from PhosR were log2 transformed for post hoc analysis with moderated t-tests using the LIMMA package and FDR correction according to Benjamini-Hochberg ^58,59^. The PhosR-processed versions of the current phosphopeptide dataset, and the Blazev et al. phosphopeptide dataset, can be found under the tabs labeled “Post PhosR” in Supplementary Tables 3 and 6, respectively, and these datasets were used for all subsequent analyses.

The `kinaseSubstrateScor` function and `kinaseSubstratePred` function within PhosR were used to predict the top three substrates for all kinases within the phosphopeptide dataset that had a known substrate in the reference database (Supplementary Software 3) ^8^. Upset plots were generated with EVenn ^60^. The Mfuzz R package was used for soft clustering and included the use of the `filter.std` function with a minimum standard deviation value of 0.1, a c-value of 4, and an m-value of 1.997 that was derived from the `mestimate`; function ^9^. Soft cluster images were created with the `mfuzz.plot2` function, and the `acore` function was used to extract the cluster membership scores for subsequent annotation of the phosphopeptide data. The phosphopeptide data was uploaded into Perseus V1.6.6.0 ^61^ for further annotation with information obtained from DAVID ^62^, and PhosphoSitePlus ^12^, and then used to perform 1D enrichment analyses within Perseus ^10^. Redundant gene ontology terms from the enrichment analyses were removed with REVIGO at the medium strength (0.7) setting ^63^. The web-based version of KSEAapp ^11^ with a NetworKIN cutoff score of 1.5 was used to infer how the different modes of exercise affected the activity of kinases. Kinases with less than 5 known or predicted substrates (i.e., m < 5) were removed, and then the output was further filtered so that only kinases present in the skeletal muscle-specific proteome/phosphoproteome datasets listed in Table 10 were retained ^55,64,65^.

### Western Blot Analysis

Frozen human and mouse muscles were homogenized for 30 sec using a Polytron in ice-cold Buffer C (40 mM Tris, pH 7.5, 1 mM EDTA, 5 mM EGTA, 0.5% Triton X-100, 25 mM β-glycerophosphate, 25 mM NaF, 1 mM Na_3_VO_4_, 10 μg/ml leupeptin, 1 mM PMSF). The whole homogenates of 3-day post-electroporation muscles were used for western blot analysis. For all other samples, the whole homogenates were centrifuged at 2,000g for 5 min at 4°C, and then the supernatant was retained for further analysis. Protein concentrations were measured with a DC protein assay kit (Bio-Rad), and equivalent amounts of protein were dissolved in Laemmli buffer, boiled for 5 min, and subjected to SDS-PAGE. Proteins were transferred to a PVDF membrane at 300 mA for 1 hr and 45 min. For total protein quantification, No-Stain^™^ (Invitrogen) was used to label the proteins on membranes before blocking as described in the manufacturer’s instructions, and total protein images were obtained by using the UV transilluminator and SYBR Gold (520–620 nm emission) filter of the UVP Autochemi system (Analytik Jena AG). Membranes were blocked with 5% powdered milk in Tris-buffered saline containing 0.1% Tween 20 (TBST) for 1 hr, except for when using the Total OXPHOS antibody, which required 5% bovine serum albumin (BSA) blocking. Details for all antibodies are provided in Supplementary Table 11. After washing in TBST for 30 min, membranes were incubated overnight at 4°C with primary antibodies dissolved in 1% BSA in TBST, except for the Total OXPHOS and P-MK2(T222) antibodies, which used 1% milk in phosphate-buffered saline (PBS) and 5% milk in TBST, respectively. The next day, membranes were washed for 30 min with TBST and probed with a peroxidase-conjugated secondary antibody in 5% powdered milk-TBST for 1 hr at room temperature. After a final 30 min of washing in TBST, blots were developed using a UVP Autochemi system with either regular enhanced chemiluminescence (ECL) reagent (Pierce) or ECL-prime (Amersham). Images were quantified using ImageJ software (U.S. NIH).

### Animals and Ethical Approval

All animal experiments followed the guidelines approved by the Institutional Animal Care and Use Committee of the University of Wisconsin-Madison (#V005375) or the William S. Middleton Memorial Veterans Hospital (Assurance ID: D16–00403). MetRS^L274G +/−^ mice were generated through a multi-step breading approach in which floxed STOP GFP-2A-MetRS^L274G^ mice (Jackson Laboratory, strain #028071) ^34^ were initially crossed with mice that express CMV-Cre mice (Jackson Laboratory, strain #006054). The presence of CMV-Cre resulted in offspring with germline cells that contained the recombined variant of the allele (i.e., it no longer contained the STOP cassette in front of the floxed STOP GFP-2A-MetRS^L274G^ transgene). The mice with germline transmission of the recombined allele (i.e., GFP-2A-MetRS^L274G^) were then crossed with wild-type C57BL/6J mice (Jackson Laboratory, strain #00664) to create offspring that ubiquitously expressed GFP-2A-MetRS^L274G^ in the absence of Cre. Offspring that were heterozygous for GFP-2A-MetRS^L274G^ were used for the experiments in this study and were referred to as MetRS^L274G +/−^ mice. All mice were housed under controlled conditions (25 °C, 12 hr light/dark cycle with lights off at 6:00 PM) and provided standard rodent chow (5001 Rodent Laboratory Chow), and water *ad libitum* unless otherwise stated. Experimental interventions were performed on male mice that were 9–10 weeks of age at the start of the intervention. MetRS^L274G +/−^ mice were utilized for the 3 hr post-training and 3-day post-electroporation interventions. All other interventions utilized wild-type C57BL/6J mice. All mice within a given intervention were randomly assigned to an experimental group and, when needed, euthanasia was performed with cervical dislocation on mice that were anesthetized with 2–5% isoflurane in oxygen.

### Mouse Model of Resistance Exercise - Weight Pulling

#### Acclimation

All mice were provided with one acclimation session between 9:00 AM and 12:00 PM for the single bout group, and between 6:00 – 9:00 PM for the long-term adaptations group, as previously described ^33^. During this session, an unweighted cart was attached to the tail and then the mice were placed at the start line on the weight pulling track and familiarized with pulling the cart to the end of weight pulling lane. At the end of the lane, the mice encountered a resting house and, upon reaching the resting house, they were given 1 min to recover before being returned to the start line on the weight pulling track. Initially, the mice were motivated to pull the unweighted cart by touching their rear fur with a wire brush close to the lumbar vertebrae region. If the mice failed to make forward progress after being touched 3 times (with a 1 s interval between each touch), then an additional incentive was provided by delivering a 1 mA shock to the lumbar vertebrae region/tail (Precision Animal Shocker, Coulbourn Instruments). The familiarization process was repeated a minimum of 8 times, and until the mouse voluntarily traversed the entire length of the weight pulling lane three consecutive times.

#### Maximal load test

The maximal load test was initiated two days after the completion of the acclimation session, between 9:00 AM and 12:00 PM for the single bout group, and between 6:00 – 9:00 PM for the long-term adaptations group. The test was aimed at determining the mouse’s maximal pulling load and consisted of ~10–15 sets with 2 min of rest between each set. During this procedure, the cart was attached to the tail and the mice were motivated to pull the cart by touching their rear fur. If the mice did not make forward progress after being touched 3 times (with an ~3 s interval between each touch), an additional 1 mA shock was delivered. The first set during this session consisted of pulling the unweighted cart and then the next three sets consisted of pulling the weighted cart with a total load of 300, 600, and 750 g. After completing these sets, an additional 50 g was added to the weighted cart and the mouse completed another set. The process of adding 50 g to the weighted cart was repeated until a load was reached during which the mouse failed to traverse the entire length of the weight pulling lane. Failure during this session was defined as the inability of the mouse to make forward progress after 3 touches and 1 shock. Upon reaching failure, assistance (i.e., gentle pushing of the cart in a manner that would be analogous to a spotter helping a human complete a final repetition during resistance exercise) was provided so that the mouse could traverse the remaining length of weight pulling lane without the need for a further touch/shock incentive. At this point, the maximal load test was complete, and the highest load successfully pulled along the entire length of the lane was defined as the mouse’s maximal pulling load.

#### Single bout for assessing changes in protein phosphorylation and myofibrillar protein synthesis

Starting at 7:00 – 10:00 AM three days after the maximal load test, the mice were fasted for 2 hr, and then one cohort was subjected to a bout of weight pulling (WP) while the other cohort was subjected to a bout of the mock/control condition (See schematics in Fig. 7a and Extended Fig 8a). For WP, the training session began with a warm-up set that only involved pulling the unweighted cart. External motivation consisted of touching the rear fur, and if the mice did not make forward progress after 5 touches (with a 3 s interval between each touch), an additional 1 mA shock was delivered. A total of 2 min of rest was given between each set, and the warm-up set was followed by sets with loads representing 50, 75, 85, 90, 95, and 100% of the mouse’s previous maximal pulling load, respectively. During all subsequent sets, an additional 15 g was added to the weighted cart, and this cycle was repeated until a load was reached during which the mouse failed to traverse the entire length of the weight pulling lane. Failure was defined as the inability of the mouse to make forward progress after 5 consecutive touches followed by 1 shock and then 2 additional touches. Upon reaching failure, assistance (as described above) was provided so that the mouse could traverse the remaining length of the weight pulling lane without the need for further touch/shock incentive. As a mock/control condition, mice were subjected to the same number of sets, rest periods, and touch/shock cycles as the WP mice, but they only pulled an unweighted cart. For the immediate (0 hr) post collections, the mice were anesthetized with 2–5% isoflurane in oxygen, and the FDL muscles were rapidly collected, frozen in liquid nitrogen, and stored at −80 °C for western blot analysis. For the 3 hr post collections, the mice were given an intraperitoneal (IP) injection of 400 μg/g bodyweight azidonorleucine (ANL) dissolved in 200 μl PBS immediately after the end of the WP or control bout, and then the mice were placed in a cage that contained water but no food. After 3 hr, the FDL muscles from these mice were collected as described for the 0 hr condition with one muscle being saved for western blot analysis and the other being saved for measurements of myofibrillar protein synthesis.

#### Paradigm for assessing long-term adaptations

The general procedures described above were used to study the adaptations that occur when mice perform 3 bouts of WP per week for 13 weeks. Extensive details about these procedures can be found in our original publication ^33^. Importantly, the same FDL muscles that were analyzed in the original publication were used to obtain the results that are reported in Figure 6 and Extended Figure 7.

### Mouse Model of Endurance Exercise - Treadmill Running

#### Acclimation

All mice were provided with one acclimation session between 9:00 AM and 12:00 PM for the single bout group, and between 6:00 – 9:00 PM for the long-term adaptations group. The mice were acclimated by placing them on the 15° incline treadmill (Exer 3/6, Columbus Instruments) at 0 m/min, allowing them to explore for 1–3 min, and then gradually increasing the speed to 3 m/min. The electric shock grid was set to 1 Hz, 1 mA, and a clean absorbent pad was placed under and over the treadmill. After 3 min the speed was increased to 6 m/min, 8 m/min after 5 min, 10 m/min after 7 min, and then the acclimation session was stopped after 10 min. Two days later, the mice were subjected to a second acclimation session with a warm-up at 3 m/min for 5 min. The speed was then increased by 1 m/min until reaching 12 m/min and the mice were maintained at this speed for a total of 20 min.

#### Single bout for assessing changes in protein phosphorylation and myofibrillar protein synthesis

Starting at 7:00 – 10:00 AM three days after the acclimation session, the mice were fasted for 2 hr, and then one cohort was subjected to a bout of treadmill running (TR) while the other cohort was subjected to a bout of the mock/control condition. The TR training session began with a warm-up at 3 m/min for 5 min. The speed was then increased by 1 m/min until reaching the target speed of 13 m/min. The mice were maintained at 13 m/min for a total of 40 min. External motivation consisted of touching the rear fur with a wire brush, and if the mice did not make forward progress after 5 touches within one min, the speed was decreased by 0.5 m/min for the remainder of the training session. As a mock/control condition, mice were subjected to the same number of touches as the TR mice, but they were only required to walk on the treadmill at 3 m/min for 10 min. For the immediate (0 hr) post collections, the mice were anesthetized with 2–5% isoflurane in oxygen, and the FDL muscles were rapidly collected, frozen in liquid nitrogen, and stored at −80 °C for western blot analysis. For the 3 hr post collections, the mice were given an IP injection of 400 μg/g bodyweight ANL immediately after the end of the TR or control bout, and then the mice were placed in a cage that contained water but no food. After 3 hr, the FDL muscles from these mice were collected as described for the 0 hr condition with one muscle being saved for western blot analysis and the other being saved for measurements of myofibrillar protein synthesis.

#### Paradigm for assessing long-term adaptations

Three days after the second acclimation session, mice were divided into two cohorts with one trained with 65 bouts of TR and the other trained with 65 bouts of a mock/control condition. All training sessions were conducted between 6:00 – 9:00 PM. For TR, each session began with a 5-min warm-up at 3 m/min, followed by a speed increase of 1 m/min until the target speed was reached. Mice were maintained at the target speed for the target duration, with sessions conducted 5 times per week for a total of 13 weeks. Both target speed and target duration were gradually increased over the 13 weeks. During the first week, the target speed increased from 12 m/min to 14 m/min, and the target duration increased from 30 min to 40 min. Over the next two weeks, the target duration increased by 10 min per week while the speed was maintained at 14 m/min. Subsequently, the target duration was maintained at 60 min while the target speed increased by 0.5 m/min every two weeks. External motivation during the first week included touching the rear fur with a wire brush and a 1 Hz electric shock grid; after the first week, only the wire brush was used. In the mock/control condition, mice were subjected to the same number of touches with the wire brush but were only required to walk on the treadmill at 3 m/min for 10 min. Immediately before starting the final week of training, grip strength was assessed for all mice using a dual-range force sensor (Vernier), as previously described ^33^.

#### Collection procedure for long-term adaptations

Terminal collections were performed 60–96 hr after the final training session. During this procedure, all potentially identifiable information (e.g., tail markings, etc.) was masked, and then the animals were weighed and subsequently anesthetized with 2–5% isoflurane in oxygen. The flexor digitorum longus, plantaris, soleus, lateral head of the triceps brachii, and long head of the triceps brachii muscles from both the left and right hindlimbs were removed, weighed, submerged in optimal cutting temperature compound (OCT, Tissue-Tek) at resting length, frozen in liquid nitrogen-chilled isopentane for 90 sec, and stored at −80 °C. At this point, the mice were euthanized by cervical dislocation, and additional skeletal muscles from both the left and right side of the body were collected, including the gastrocnemius, pectoralis major, and the forearm flexor complex. In addition to skeletal muscles, tissues including the heart, the left and right tibias, epididymal fat pads, interscapular brown adipose tissue, and adrenal glands were also collected. Importantly, all of the collection procedures were performed by blinded investigators.

### In-gel visualization and quantification of newly synthesized proteins

Frozen FDL muscles were immersed in 10 μL/mg of ice-cold Buffer D (67 mM sucrose, 50 mM Tris/HCl, 50 mM KCl, 12 mM EDTA, pH 7.4, plus phosphatase and protease inhibitors including 25 mM NaF, 15 mM β-glycerophosphate, 10 μg/ml leupeptin, 1 mM Na_3_VO_4_, 10 μg/ml aprotinin, 1 μg/ml pepstatin, and 1 mM PMSF). The tissue was then minced with scissors and subsequently homogenized with an ice-cold glass Dounce homogenizer. The lysate was centrifuged at 700g for 10 min at 4°C, and the supernatant volume was measured prior to its removal. The remaining myofibrillar protein pellet was washed by resuspending it in 500 μL of Buffer D and then centrifuging at 700g for 15 min. The pellet was washed twice and then resuspended in Buffer D using the same volume as the volume of supernatant that was originally removed. The protein concentration of the resulting sample was then measured with a DC protein assay kit (Bio-Rad). For the DBCO-based ‘click’ reaction, 134 μg of protein was precipitated with ice-cold acetone (4:1 volume to volume ratio), centrifuged at 12,000g for 5 min, and then the acetone was removed. The pellet was air-dried for 5 min and subsequently resuspended in 90 μL of freshly prepared Buffer E (8 M urea, 50 mM Tris (pH 8), 100 M 2-chloroacetamide, plus phosphatase and protease inhibitors including 25 mM NaF, 15 mM β-glycerophosphate, 10 μg/ml leupeptin, 1 mM Na_3_VO_4_, and 1 mM PMSF). To assist with resuspension, the pellet was subjected to 50–100 strokes with a microfuge pellet pestle (Kimble) and continuously vortexed until the pellet had completely dissolved (~1–5 min). Once the pellet had dissolved, the ANL-labeled proteins were tagged with Cy5.5 by adding 10 μL of a DBCO stock solution (134 μM Cy5.5 DBCO (Click Chemistry Tools) dissolved in PBS containing 40% DMSO). The combined solution was protected from light and incubated on a rotator (9 rpm) for 1 hr at room temperature. Following the incubation, the proteins were precipitated via the addition of 900 μL of 100% methanol and then centrifuged at 12,000g for 5 min. The supernatant was removed, and the pellet was washed by resuspending in 900 μL of 100% methanol and centrifuging at 12,000g for 5 min. The supernatant was again removed, and the pellet was air-dried for 5 min and then resuspended in 100 μL of Buffer C containing 2% SDS. The resulting sample was dissolved in Laemmli buffer, boiled for 5 min, and subjected to SDS-PAGE at 100 V until the dye front was 5 mm from the bottom of the gel. The manufacturer instructions for No-Stain^™^ (Invitrogen) were then used to label all proteins in the gel, and the gel was imaged with the universal mode of an iBright^™^ FL1500 (Invitrogen) and the default filters for a No-Stain labeled gel as well as Alexa Fluor 680 (610–660 nm excitation, 710–730 nm emission) for the visualization of the Cy5.5 (i.e., the ANL-labeled proteins). Images were quantified using ImageJ software (U.S. NIH).

### Histological imaging and analysis of the FDL muscles

Mid-belly cross-sections (10 μm thick) of FDL muscles that had been frozen in OCT were obtained with a −20°C cryostat (Leica) and subsequently fixed for 10 min with −20°C acetone. A hydrophobic circle was drawn around each section using a PAP pen (Thermo Fisher Scientific) and then the sections were washed with PBS for 15 min (3 × 5 min) and blocked for 20 min at room temperature in Buffer F (0.5% Triton X-100, 0.5% BSA in PBS). For fiber type staining, the blocked samples were incubated for 1 hr at room temperature in Buffer F containing rabbit anti-laminin, mouse IgG2b anti-Type I MHC, mouse IgG1 anti-Type IIA MHC, and mouse IgM anti-Type IIB MHC. The sections were then washed three times for 15 min (3 × 5 min) with PBS and incubated for 1 hr at room temperature in Buffer F containing Alexa 568 goat anti-rabbit IgG, Alexa 647 goat anti-mouse IgG2b, Alexa 488 goat anti-mouse IgG1, and Alexa 350 goat anti-mouse IgM. For capillary staining, the blocked samples were incubated for 1 hr at room temperature in Buffer F containing rat anti-CD31 and rabbit anti-laminin. The sections were then washed three times for 15 min (3 × 5 min) with PBS and incubated for 1 hr at room temperature in Buffer F containing Alexa 488 goat anti-rat and Alexa 568 goat anti-rabbit IgG. Following the incubation with the secondary antibodies, the sections were washed with PBS for 15 min (3 × 5 min), mounted in ProLong Gold anti-fade mounting medium (Invitrogen), and overlaid with a coverslip (Thermo Fisher Scientific). Entire muscle cross-sections were then imaged by a blinded investigator with a 10X objective on a BZ-X700 Keyence microscope with four different filters (DAPI, GFP, TRITC, CY5) for fiber type staining and two different filters (GFP, TRITC) for capillary staining. From these images, fiber type-specific measurements of fiber CSA and the proportion of each fiber type within each section were determined with our automated “fiber cross-sectional area” CellProfiler pipeline as previously described ^33^. For measurements of the number of capillaries per fiber, a modification of our previously published myonuclei CellProfiler pipeline was employed (Supplementary Software 4) ^33^. Details for all antibodies are provided in Supplementary Table 11

### Plasmid DNA and electroporation

Plasmid DNA encoding LacZ has been previously described ^66^. Plasmids encoding c.a. MKK3b, c.a. MKK4, c.a. MKK6, and tdTomato were obtained from Addgene (catalog numbers: 50449, 14813, 13518, and 54642, respectively). All plasmid DNA was propagated in DH5α E. coli, purified using an EndoFree Plasmid Kit (QIAGEN), and re-suspended in sterile phosphate-buffered saline (PBS). Electroporations were conducted with modifications of our previously described methods ^38^. Specifically, mice were anesthetized with 2–5% isoflurane in oxygen, and a small incision was made through the skin overlying the TA muscle. A 27-gauge needle was used to inject 12 μL of a DNA solution into the TA muscle. The solution contained 30 μg of plasmid DNA encoding one of the following: c.a. MKK3b, c.a. MKK4, c.a. MKK6, or LacZ, along with 3 μg plasmid DNA encoding tdTomato when indicated. Subsequently, electric pulses were administered to the muscle via a 1 cm gap pin electrode (Harvard Apparatus) that was laid across the proximal and distal myotendinous junctions. Eight 20 ms square-wave electric pulses at a frequency of 1 Hz were delivered using an ECM 830 electroporation unit (Harvard Apparatus) at a field strength of 160 V/cm. Following electroporation, the incision was closed with sutures and Vetbond surgical glue (3M Animal Care Products), and the animals were given an IP injection of 0.05 μg/g buprenorphine to mitigate any pain. After a 3- or 7-day recovery period, the mice were anesthetized with 2–5% isoflurane in oxygen, and the TA muscles were either immediately removed and frozen in liquid nitrogen for western blot analysis or collected following perfusion fixation as described below. Where indicated, the mice were also given an IP injection of 400 μg/g bodyweight of ANL at 3 hr prior to the collection.

### Perfusion fixation and cryoprotection

Silicone tubing was connected to a peristaltic pump with one end of the tubing connected to a 23-gauge needle (Sol-Millennium Medical Inc.) while the other end was inserted into a flask containing 30 mL per mouse of ice-cold Buffer G (PBS, 25 mM β-glycerophosphate, 25 mM NaF, 1 mM Na_3_VO_4_) and the tubing was cleared of air bubbles. Mice were weighed and anesthetized using a 300 mg/kg ketamine and 30 mg/kg xylazine mixture, verifying complete anesthesia by checking corneal and pedal pain reflexes. If needed, further anesthesia was induced with an IP injection of 50 mg/kg ketamine or via 2% isoflurane in oxygen. The mouse was placed in a supine position and their limbs were secured. Next, an incision was made into the abdominal cavity, followed by cutting through the diaphragm and ribcage to expose the heart. The needle was then inserted into the apex of the left ventricle. Perfusion began at ~1 mL/min with ice-cold Buffer G, the right atrium was then cut, and the flow was increased to 7 mL/min. Perfusion continued with all 30 mL of Buffer G, and then the perfusate was switched to 30 mL of ice-cold Buffer H (4% paraformaldehyde in 0.1 M phosphate buffer (pH 7.2), 25 mM β-glycerophosphate, 25 mM NaF, 1 mM Na_3_VO_4_). After the perfusion fixation was complete, the hindlimbs were skinned, excised from the mouse, and fixed in Buffer H for 3 hr at 4°C with orbital rocking at 50 rpm. The TA muscles were then removed and incubated overnight with light agitation in 1 mL of Buffer H at 4°C. The TA was subsequently cryoprotected by incubating it with light agitation in 1 mL of 0.1 M phosphate buffer (pH 7.2) containing 15% sucrose for 6 hr at 4°C, and then 1 mL of 0.1 M phosphate buffer (pH 7.2) with 45% sucrose for 18 hr at 4°C. The cryoprotected muscle was then embedded in ice-cold OCT (Tissue-Tek), frozen in liquid nitrogen-chilled isopentane for 90 sec, and stored at −80°C.

### Histological imaging and analysis of electroporated muscles

Mid-belly cross-sections (3 μm thick) of the TA muscles were obtained with a −35°C cryostat (Leica) and immediately dipped into distilled water. Residual water around the section was removed and a hydrophobic circle was drawn around each section using a PAP pen (Thermo Fisher Scientific). The sections were washed with PBS for 5 min and blocked for 30 min at room temperature in Buffer F. For the 3-day post-electroporation samples, the sections were washed with PBS (3 × 5 min) and then subjected to a TBTA-based ‘click’ reaction to tag ANL-labeled proteins. Specifically, a 300 μL ‘click’-reaction mixture (enough for 6 sections) was prepared by adding the following reagents in the order listed along with vortexing between each addition: (1) 277.5 μL of PBS, (2) 1.5 μL of 20 mM TBTA in DMSO, (3) 3 μL of 100 mM CuSO4 in diH2O, (4) 12 μL of 50 μM alkyne-AZDye 647 (Click Chemistry Tools) in DMSO, and (5) 6 μL of 50 mM TCEP in diH2O. Once combined, each section was incubated in 50 μL of the ‘click’-reaction mix, protected from light, and rocked at 50 rpm for 1 hr at room temperature. The sections were then subjected to alternating 5-min washes with PBS (3x) and Buffer F (3x) at room tempetaure, followed by an additional set of washes (1 × 5 min and then 1 × 20 min) in Buffer F containing 10% DMSO and 50 mM EDTA. After additional (3 × 5 min) washes with PBS, the sections were incubated overnight at room temperature with rabbit P-S6(240_4) antibody in Buffer F. The sections were then washed (3 × 5 min) and (3 × 1 hr) with Buffer F at room temperature. Each section was then incubated overnight at room temperature with goat anti-rabbit Alexa Fluor^®^ 405 Plus in Buffer F. Finally, the sections were subjected to (3 × 5 min) and then (3 × 1 hr) washes in Buffer F at room temperature, and the unmounted and wet sections were then imaged as detailed below.

The processing of 7-day post-electroporation samples utilized the same basic procedures described above, except the samples were not subjected to the TBTA-based click reaction and subsequent DMSO/EDTA washes. Furthermore, rather than probing for P-S6(240_4), the periphery of the muscle fibers was identified by subjecting the samples to overnight incubations with rabbit anti-dystrophin and goat anti-rabbit CF^®^640R in Buffer F, or a 1 hr incubation with WGA-640R in Buffer F. Following these incubations, the samples were washed as described above, incubated with Hoechst for 5 min to identify nuclei, and then washed (3 × 5 min) with PBS at room temperature. Each unmounted and wet section was imaged as described below. Details for all antibodies are provided in Supplementary Table 11.

Images of the unmounted and wet samples were collected with a 10X objective on a Leica Widefield Thunder Microscope and 405 (Ex: 405/60, EM: 470/40), Y3 (Ex: 545/26, EM: 605/70), and Y5 (Ex: 620/60, EM: 700/76) filters. Prior to image acquisition, all sample identification information was masked, and then 2–4 regions per sample with tdTomato positive fibers (i.e., transfected fibers) were identified. Images of these regions were then collected in a manner so that an approximately equal number of transfected and non-transfected fibers were present. Within each region, the periphery of 23–35 transfected fibers along with an equal number of non-transfected fibers were manually traced with NIS-Elements D software (Nikon). When selecting these fibers, care was taken to ensure that the transfected and non-transfected fibers were chosen equitably based on their proximity to the periphery of the muscle section, and fibers with centrally located nuclei were excluded from consideration. The traced muscle fibers were then used to determine fiber CSA and, when applicable, the mean intensity of the signal for P-S6(240_4) and ANL-labeled proteins.

### Statistical Analysis

All non-omic data sets were screened for within-group outliers by applying the “very conservative” three median absolute deviations (3 MAD) rule described by Leys et al., 2012 ^67^, and individual data points that exceeded the 3 MAD threshold were removed (note: the source data sets included all values that were obtained and the 3 MAD outliers are clearly highlighted). Statistical analyses on the resulting data sets were conducted using various methods to accommodate the diverse experimental designs. To account for both within-subject and between-subject variability, one-way repeated measures (RM) ANOVA or one-way mixed ANOVA followed by post hoc analysis with the two-stage step-up method of Benjamini, Krieger, and Yekutieli were employed. Two-way ANOVA was conducted to assess the main effects and interactions between two independent variables, followed by Sidak’s or Fisher’s LSD post hoc analyses. For direct comparisons between two groups, two-tailed Student’s t-tests or paired t-tests were applied. All tests adhered to a statistical significance threshold of *P* < 0.05 and were performed with GraphPad Prism version 10.2.3 for Windows.

## Supplementary Material

Supplement 1

## Data Availability

The RAW data files are available on MassIVE (https://massive.ucsd.edu/ProteoSAFe/index.jsp) under identifier MSV000093793, accessible with username: MSV000093793_reviewer and password: reviewer_pw.
