# Supplementary material for: Identification of a Resistance Exercise-Specific Signaling Pathway that Drives Skeletal Muscle Growth
Source: Res Sq. 2024 Nov 12:rs.3.rs-4997138. Preprint. [Version 1] doi: 10.21203/rs.3.rs-4997138/v1 (PMC11601848; doi:10.21203/rs.3.rs-4997138/v1)
Supplement: Supplement 1 [file NIHPPRS4997138V1-supplement-1.pdf]

Extended Figure Legends

Extended Figure 1. Gene ontology enrichment in phosphopeptide clusters 1 and 2
1D enrichment analysis of gene ontology (GO) terms when using the membership score of the phosphopeptides for "cluster 1" (**a**), or "cluster 2" (**b**) as defined in Figure 2. All GO terms were assigned a rank-based score between -1 and 1, with a negative score indicating under-representation of the term and a positive score indicating over-representation. Redundant GO terms were then removed with REVIGO. In the graphs, the score for each term was plotted against the $-\mathrm{Log}_{1}$ of its respective *q*-value. The displayed dots indicate GO terms with a *q*-value of < 0.05. GO terms of interest are highlighted in each graph, and a full list of the outcomes is provided in the source data table.

Extended Figure 2. Machine learning-based prediction of kinase-substrate relationships
Clustered heatmap of the kinases-substrate score for the top three phosphorylation sites of all kinases with a known substrate in the phosphopeptide dataset. A higher score indicates a higher likelihood of being a substrate for the listed kinase. The top three sites for MAPKAPK2 are highlighted in the green box.

Extended Figure 3. Reproducibly of the endurance and resistance exercise-induced changes in the phosphorylation of MAPKAPK substrates
Heatmaps of the mean exercise-induced change in the phosphorylation of the known and/or predicted substrates of the MAPKAPK's that were identified in both the current study and the phosphopeptide dataset of Blazev et al., 2022 [14]. Also shown is the number of participants (n) that the mean values were obtained from in each dataset.

Extended Figure 4. Quantitative results from the western blots in Figure 4
Biopsies from the experimental interventions described in Figure 4c were subjected to western blot analysis as shown in Figure 4d. **a**-**k**, For each participant, the phospho to total protein ratio (P/T) for the indicated signaling event in each biopsy was determined and then expressed relative to the mean value observed in the pre-exercise biopsies. Values in the graphs are presented as the group mean ± SEM, n = 9-12 per group. The data was analyzed with one-way mixed ANOVA. * Significantly different from Pre, † 0 hr END vs. 0 hr RE, ‡ 3 hr END vs. 3 hr RE, $P < 0.05$.

Extended Figure 5. Relationship between the exercise-induced changes in myofibrillar protein synthesis and the phosphorylation state of various signaling molecules
Linear regression was used to compare each participant's endurance and resistance exercise-induced change (Δ) in myofibrillar protein synthesis (MyoPS) with the change in the phospho to total protein ratio (P/T) for each of the signaling events analyzed in Figure 4. **a**,**b**, Signaling data from the 0 hr post-exercise time-point. **c**,**d**, Signaling data from the 3 hr post-exercise time-point. **a**,**c**, Multivariable plots that illustrate the coefficient of determination (R-squared) and *P*-value of the co-relationship for all comparisons that revealed an R-squared value of >0.1. NS equals not significant. **b**,**d**, Graphs of the co-relationships that exist between the Δ in MyoPS and the Δ in the P/T for MKK3(S218). Individual values for each participant were expressed as a percentage of the value obtained in their respective pre-exercise sample. Dashed lines represent the 95% confidence intervals.

Extended Figure 6. Long-term adaptations in the mouse model of endurance exercise
Mice were subjected to 13 weeks of training with treadmill running (TR) or a mock (control) paradigm. The average weekly (**a**) body weight, and (**b**) workload per training session, as well as (**c**) the number of times the rear of the animal was touched during each training session. Individual data points are displayed with hollow symbols and the weekly means for each group are displayed with solid symbols. **d**-**r**, After 13 weeks of training, the mice were subjected to measurements of (**d**) grip strength, and (**e**) tibia length (TL). The mass of the (**f**) individual epididymal (Epi.) fat pads, (**g**) interscapular brown adipose tissue (iBAT), (**h**) adrenal glands, and (**i**) heart were measured and normalized to TL. **j**, The mass of individual muscles (MM) including the gastrocnemius (GAST), plantaris (PLT), soleus (SOL), flexor digitorum longus (FDL), pectoralis major (PEC), triceps brachii lateral head (Tri-Lat), triceps brachii long head (Tri-Long), and the forearm flexor complex (FF) were all normalized to TL and expressed relative to the mean value observed in the control group. **k**, Mid-belly cross-

sections of the FDL muscles were subjected to immunohistochemistry (IHC) for laminin and fiber type identification (i.e., Type I, IIA, IIX, or IIB), scale bars = 500 µm. The entire cross-section was used to determine (**l**), the average cross-sectional area (CSA) of the different fiber types, and (**m**) the proportion of the fibers that were represented by each fiber type. **n**, Mid-belly cross-sections of the FDL muscles were subjected to IHC for laminin and CD31 to identify capillaries, scale bars = 25 µm. **o**, The entire cross-section was used to determine the average number of capillaries per fiber. **p-r**, FDL muscles were subjected to western blot analysis for (**q**) members of the five OXPHOS complexes (i.e., CI - CV), and (**r**) other mitochondrial (mito.) proteins. For each sample, the individual protein content was normalized to the total amount of protein loaded on the gel and then expressed relative to the mean of the control group. Values in the graphs are presented as the group mean ± SEM, n = 6-10 per group. The data were analyzed with two-way repeated measures (RM) ANOVA (**a**), one-way RM ANOVA (**b,c**), paired t-tests (**d-i**, and **o**), or two-way ANOVA (**j**, **l**, **m**, **q**, **r**). ■ Significantly different from week 1, or * the control group, $P < 0.05$.

Extended Figure 7. Long-term adaptations in the mouse model of resistance exercise
Flexor digitorum longus (FDL) muscles were collected from mice that had completed 13 weeks of training with weighted pulling (WP) or an unweighted (control) paradigm as previously reported by Zhu et al. 2021 [33]. **a**, Mid-belly cross-sections of the FDL muscles were subjected to immunohistochemistry for laminin and CD31 to identify capillaries, scale bars = 25 µm. **b**, The entire cross-section was used to determine the average number of capillaries per fiber. **c-e**, FDL muscles were subjected to western blot analysis for (**d**) members of the five OXPHOS complexes (i.e., CI - CV), and (**d**) other mitochondrial (mito.) proteins. For each sample, the individual protein content was normalized to the total amount of protein loaded on the gel and then expressed relative to the mean of the control group. Values in the graphs are presented as the group mean ± SEM, n = 9-10 per group. The data were analyzed with paired t-tests (**b**)**,** or two-way ANOVA (**d**, **e**).

Extended Figure 8. A rapid and robust activation of signaling through MKK3/4/6, p38, and MK2 occurs specifically in response to resistance exercise in mice
a, Schematic of how C57BL6 mice were subjected to endurance exercise with treadmill running (TR), resistance exercise with weight pulling (WP), or their respective mock-trained (control) conditions. **b**, FDL muscles from the mice were collected immediately after the last training bout and subjected to western blot analysis for the phospho (P) and total (T) levels of the indicated proteins. Long isoform of MK2 (L), short isoform of MK2 (S). **c**, For each sample, the phospho to total protein ratio (P/T) for each signaling event was determined and expressed relative to the mean value observed in the treadmill control group. Values in the graphs are presented as the group mean ± SEM, n = 4-5 per group. The data were analyzed with two-way ANOVA, * significantly different from the TR control group, or † the weight pulling control group, $P < 0.05$. ■ Significant difference between the TR control and TR trained group when the planned comparison was analyzed with a Student's t-test, $P < 0.05$.

Extended Figure 9. Quantitative results from the western blots in Figure 7
FDL muscles from the experimental conditions described in Figure 7a were subjected to western blot analysis as shown in Figure 7e. **a-k**, For each sample, the phospho to total protein ratio (P/T) for each signaling event was determined and then expressed relative to the mean value observed in the treadmill (TR) control group. Values in the graphs are presented as the group mean ± SEM, n = 4-5 per group. The data were analyzed with two-way ANOVA. Significantly different from * the TR control group, or † the weight pulling (WP) control groups, $P < 0.05$.

Extended Figure 10. Quantitative results from the western blots in Figure 8
TA muscles were subjected to western blot analysis as described in Figure 8b. **a-i**, For each sample, the total (T) protein level, or the phospho to total protein ratio (P/T) for the indicated signaling event, was determined and then expressed relative to the mean value observed in the LacZ control group. Values in the graphs are presented as the group mean ± SEM, n = 4-5 per group. The data were analyzed with one-way ANOVA. Significantly different from * LacZ, or † c.a. MKK3b, $P < 0.05$.

Supplementary Information

Supplemental Figure 1: Constitutively active MKK4 does not induce signaling through its canonical substrates in electroporated tibialis anterior muscles

Supplementary Table 1: Characteristics of the participants in the proteomics/phosphoproteomics study

Supplementary Table 2: Proteomics dataset

Supplementary Table 3: Pre- and Post-PhosR versions of the phosphopeptide dataset

Supplementary Table 4: 1D enrichment analysis of clusters 1 and 2

Supplementary Table 5: KSEAapp-based inferences of kinase activity and the kinase-substrate links derived from the Post-PhosR dataset in Supplementary Table 3

Supplementary Table 6: Pre- and Post-PhosR versions of the Blazev et al. 2022 phosphopeptide dataset

Supplementary Table 7: KSEAapp-based inferences of kinase activity and the kinase-substrate links derived from the Post-PhosR dataset in Supplementary Table 6

Supplementary Table 8: KSEAapp-based inferences of kinase activity derived from the 3 hr post RE vs. 3 hr post END Post-PhosR phosphopeptide data in Supplementary Tables 3 and 6

Supplementary Table 9: Characteristics of the participants in the follow-up study.

Supplementary Table 10: Compendium of proteins that have been identified in skeletal muscle

Supplementary Table 11: Detailed description of the antibodies used in the study

Code Availability

Supplementary Software 1: The modified PhosR programming code in R language that was used to remove batch effects and generate the Supplementary Table 3 Post-PhosR dataset.

Supplementary Software 2: The modified PhosR programming code in R language that was used to remove batch effects and generate the Supplementary Table 6 Post-PhosR dataset.

Supplementary Software 3: The modified PhosR programming code in R language that was used to generate the substrate kinase scores shown in Extended Figure 2.

Supplementary Software 4: The CellProfiler pipeline that was used to determine the capillary density in whole muscle cross-sections.

Supplementary Files

This is a list of supplementary files associated with this preprint. Click to download.

- SupplementaryTable1PhosphoproteomicsStudyParticipantCharacteristics.xlsx
- SupplementaryTable2ProteomicsDataset.xlsx
- SupplementaryTable3PhosphopeptideDataset.xlsx
- SupplementaryTable41DEnrichmentAnalysisforClusters1and2.xlsx
- SupplementaryTable5KSEAappInferencesandKinaseSubstrateLinks.xlsx
- SupplementaryTable6PhosphopeptideDatasetBlazev.xlsx
- SupplementaryTable7KSEAappInferencesandKinaseSubstrateLinksfromBlazevPhosphopeptideData.xlsx
- SupplementaryTable8KSEAapp3hrREvs3hrEND.xlsx
- SupplementaryTable9FollowupStudyParticipantCharacteristics.xlsx
- SupplementaryTable10CompendiumofProteinsthathavebeenIdentifiedinSkeletalMuscle.xlsx
- SupplementaryTable11AntibodyList.xlsx
- SupplementalFigure1.jpg
- ExtendedFigure1.jpg
- ExtendedFigure2.jpg
- ExtendedFigure3.jpg

- ExtendedFigure4.jpg
- ExtendedFigure5.jpg
- ExtendedFigure6.jpg
- ExtendedFigure10.jpg
- ExtendedFigure7.jpg
- ExtendedFigure4.jpg
- ExtendedFigure8.jpg
- ExtendedFigure9.jpg
- ExtendedFigure10.jpg
